# Review: optimizing genomic selection for crossbred performance by model improvement and data collection

**DOI:** 10.1093/jas/skab205

**Published:** 2021-07-05

**Authors:** Pascal Duenk, Piter Bijma, Yvonne C J Wientjes, Mario P L Calus

**Affiliations:** Animal Breeding and Genomics, Wageningen University and Research, P.O. Box 338, 6700 AH Wageningen, The Netherlands

**Keywords:** accuracy, crossbreeding, crossbred performance, genomic prediction, genomic selection, response to selection

## Abstract

Breeding programs aiming to improve the performance of crossbreds may benefit from genomic prediction of crossbred (CB) performance for purebred (PB) selection candidates. In this review, we compared genomic prediction strategies that differed in 1) the genomic prediction model used or 2) the data used in the reference population. We found 27 unique studies, two of which used deterministic simulation, 11 used stochastic simulation, and 14 real data. Differences in accuracy and response to selection between strategies depended on i) the value of the purebred crossbred genetic correlation ($r_{pc}$), ii) the genetic distance between the parental lines, iii) the size of PB and CB reference populations, and iv) the relatedness of these reference populations to the selection candidates. In studies where a PB reference population was used, the use of a dominance model yielded accuracies that were equal to or higher than those of additive models. When $r_{pc}$ was lower than ~0.8, and was caused mainly by G × E, it was beneficial to create a reference population of PB animals that are tested in a CB environment. In general, the benefit of collecting CB information increased with decreasing $r_{pc}$. For a given $r_{pc}$, the benefit of collecting CB information increased with increasing size of the reference populations. Collecting CB information was not beneficial when $r_{pc}$ was higher than ~0.9, especially when the reference populations were small. Collecting only phenotypes of CB animals may slightly improve accuracy and response to selection, but requires that the pedigree is known. It is, therefore, advisable to genotype these CB animals as well. Finally, considering the breed-origin of alleles allows for modeling breed-specific effects in the CB, but this did not always lead to higher accuracies. Our review shows that the differences in accuracy and response to selection between strategies depend on several factors. One of the most important factors is $r_{pc}$, and we, therefore, recommend to obtain accurate estimates of $r_{pc}$ of all breeding goal traits. Furthermore, knowledge about the importance of components of $r_{pc}$ (i.e., dominance, epistasis, and G × E) can help breeders to decide which model to use, and whether to collect data on animals in a CB environment. Future research should focus on the development of a tool that predicts accuracy and response to selection from scenario specific parameters.

## Introduction

Crossbreeding is the practice of mating animals from different purebred (PB) lines to produce crossbred (CB) animals, and is widely applied in pig and poultry production. This practice allows breeders to benefit from breed complementary by selecting parental lines for different traits and combine these traits in the crossbreds (Smith, 1964). In addition, crossbreeding enables breeders to capitalize on heterosis, which is the phenomenon of superior average performance of crossbreds compared with the average performance of their parental lines (Dickerson, 1973).

The aim of CB breeding programs is to improve the performance of crossbreds, because crossbreds are the production animals. However, selection takes place in the PB parental lines, and is typically based on PB performance. Selection on PB performance usually generates a response to selection in CB performance as well, because the genetic correlation between PB and CB performance ($r_{pc}$) is generally positive, with most estimates ranging from 0.5 to 1 (Wei and van der Werf, 1995; Lukaszewicz et al., 2015; Mulder et al., 2016; Wientjes and Calus, 2017; Duenk et al., 2019a). PB and CB performance, however, are genetically not the same (i.e., $r_{pc}$ is lower than 1) as a result of genotype by environment interactions (G × E) (Falconer, 1952; Lutaaya et al., 2001), and genotype by genotype interactions (G × G) in combination with allele frequency differences between parental lines (Wei et al., 1991; Baumung et al., 1997; Duenk et al., 2021).

Because $r_{pc}$ is generally smaller than one, the response to selection in CB performance may be increased when selecting for CB performance directly, instead of relying on a correlated response from selection on PB performance. This strategy requires that PB selection candidates have estimated breeding values (estimated BV, or EBV) for CB performance. Such EBV can be obtained by measuring performance (i.e., phenotypes) of CB animals that are related to the PB selection candidates, and traditionally requires that their pedigree is known. In practice, however, phenotypes of closely related CB animals may not be available at the time of selection, and the pedigree of CB animals is often not recorded.

The need for a recorded pedigree can be alleviated by estimating BV for CB performance using genomic prediction, resulting in genomic EBV (GEBV). Genomic prediction makes use of a so-called reference population that consists of individuals that have marker genotype and phenotype information available to estimate GEBV of selection candidates that only have marker genotype information available (Meuwissen et al., 2001). With this strategy, breeders can use a reference population of CB animals to estimate GEBV for CB performance in PB selection candidates. The benefits of using CB information in genomic prediction may be that 1) response in CB performance does not rely on $r_{pc}$, 2) pedigree information of the CB animals is not required, and 3) the CB animals with phenotypes do not have to be as closely related to the PB selection candidates as would be required when relying on pedigree information.

In most breeding programs, PB selection candidates are already genotyped and phenotyped, to enable accurate selection among them based on GEBV. Naturally, this information contributes to the reference population. Phenotyping and genotyping CB animals in addition to PB animals involve making additional costs that should be offset by commercial benefits, such as increased market share. It is, therefore, important for breeders to be able to predict the benefits of phenotyping or genotyping CB animals beforehand, so that they can decide whether or not to collect such data. To date, several studies have compared accuracy of GEBV and response with selection of strategies that differ in the data or model that was used to estimate GEBV. However, a clear overview of these comparisons is lacking.

This review focuses on strategies to estimate BV for CB performance of PB selection candidates using genomic prediction. Recently, Stock et al. (2020) reviewed genomic models for the analysis of CB data, where the focus was on the differences in parameterizations between the models. Although they discussed the benefits of some models over others, they did not provide an extensive comparison between models for accuracy and response to selection. Here, we will focus on the advantages of 1) improving the genomic prediction model, and of 2) collecting data on CB animals. The strategies will be evaluated based on prediction accuracy and response to selection. First, we will discuss some theory to clearly define BV for CB performance and we discuss their estimation. Second, we will describe the studies included in this review, and the information that we extracted from them. Third, based on the included studies, we will discuss different strategies to estimate BV for CB performance of genotyped PB selection candidates. In the discussion of strategies, we will start with a “baseline strategy” that uses only PB data and a simple additive genomic prediction model. Then we move on to alternative strategies that differ in the genomic prediction model, or in the type of data used. For each strategy, we discuss its expected advantages and disadvantages based on theory, followed by a literature review of studies that compared these strategies with alternative strategies. We summarize our findings for each strategy in concluding paragraphs at the end of the respective section. Finally, we give some recommendations and practical guidelines that could be helpful for breeders to decide whether or not to collect data on CB animals.

### Theory on genomic estimated breeding values for CB performance

This review article focuses on the estimation of BV for CB performance of animals in PB parental lines. Such BV can be defined as


$$\begin{array}{l} u_{CB}=Z_{PB}\alpha_{CB}^{Q}, \end{array}$$ (1)

where $Z_{PB}$ is a matrix containing genotypes (i.e., allele counts coded as 0, 1, or 2) of PB selection candidates at quantitative trait loci (QTL), and $\alpha_{CB}^{Q}$ is a vector of average effects for CB performance at QTL in the PB line. In reality, $u_{CB}$ remains unknown because the QTL genotypes and average effects of QTL are unknown. Instead, we will have to rely on marker information to estimate $u_{CB}$ with genomic prediction. The assumption is that the markers are in linkage disequilibrium (LD) with the QTL, and that as a result, the markers capture at least part of the effects at QTL. The GEBV can be defined as


$$\begin{array}{l} {\hat{u}}_{CB}=M_{PB}{\hat{\alpha }}_{CB}, \end{array}$$ (2)

where $M_{PB}$ is a matrix containing genotypes of PB selection candidates at markers, and ${\hat{\alpha }}_{CB}$ is a vector of estimated average effects for CB performance at those markers. Genomic prediction uses a reference population to estimate the average effects of the markers, after which estimated BV of genotyped selection candidates can be computed using equation (2).

The response to selection in CB performance depends on the accuracy of ${\hat{u}}_{CB}$ ($\rho_{CB}$), measured as the correlation between ${\hat{u}}_{CB}$ and $u_{CB}$. Factors that influence $\rho_{CB}$ can be determined by comparing equations (1) and (2) (Daetwyler et al., 2008). First, ${\hat{u}}_{CB}$ are based on genotypes at markers, while $u_{CB}$ are based on genotypes at QTL. The $\rho_{CB}$, therefore, depends on how much of the genetic variation at the QTL is captured by the markers, which is a function of the strength of LD between markers and QTL. In addition, $\rho_{CB}$ depends on how well allele frequencies and LD between markers and QTL in the reference population resemble those in the selection candidates. Second, ${\hat{u}}_{CB}$ are based on estimated effects at markers (${\hat{\alpha }}_{CB}$). The $\rho_{CB}$^,^ therefore, depends on how accurate ${\hat{\alpha }}_{CB}$ are estimated. Both these factors are affected by the type of data in the reference population, and by the genomic prediction model used.

In the following sections, we start with describing the requirements for studies to be included in our review, and following those, we discuss strategies to estimate ${\hat{\alpha }}_{CB}$ (and subsequently ${\hat{u}}_{CB}$) that differ in the genomic prediction model or in the type of data.

### Criteria for inclusion of results of studies

In this review, we included results of studies that met the following criteria:

- estimated breeding values for CB performance of PB selection candidates with genomic prediction
- reported accuracy of breeding values for CB performance of PB animals, or response to selection in CB performance
- compared at least two strategies that differ in the genomic prediction model or the type of data

In total, we found 29 studies that fulfilled these criteria. All studies involved data of pigs or poultry, or simulations aimed at resembling data of these species. The strategies discussed in these studies can differ in the genomic prediction model that is applied or the type of data that is used. We evaluated each strategy by comparing its $\rho_{CB}$ (hereafter called “accuracy”) or response to selection in CB performance with a base strategy. In the comparisons of two strategies in this review, the following conditions are met, unless otherwise mentioned:

- the two strategies have similar reference population sizes
- the two strategies have the same (or similar) relationship between animals in the reference population and the selection candidates
- accuracies are correlations between GEBV and true BV for CB performance of purebreds (with simulations), or obtained with cross-validation in purebreds (with empirical data). The validation record used with cross-validation is based on (deregressed) breeding values for CB performance of purebreds.

We have excluded strategies where validation was performed at the level of the individual CB animals, the reference population consisted of CB animals, and the breed-origin of alleles in crossbreds was not accounted for. In those strategies, accuracies were correlations between GEBV and true BV of CB animals (with simulations), or correlations between GEBV and corrected phenotypes of CB animals (in empirical data). Such strategies were excluded because they may overestimate the accuracy of GEBV in purebreds (Duenk et al., 2019b), and were found in Hidalgo et al. (2015), Lopes et al. (2017), Pocrnic et al. (2019), Duenk et al. (2019b), and Alvarenga et al. (2020). After excluding these strategies, there were 27 unique studies left, two of which used deterministic simulation, 11 used stochastic simulation, and 14 used real data.

For each comparison of strategies, we present the results in a table. The column names with descriptions and abbreviations are given in Table 1. We do not aim to present a comprehensive list of all comparisons, but rather a clear overview of the most important results. For that purpose, we excluded some comparisons from large studies, and between strategies that differed markedly from reality (such as strategies with extremely low marker densities or small reference populations).

**Table 1.** Description of column names and abbreviations used in tables of comparisons between strategies

Column name	Description	Abbreviations used
Study	Last name of the first author and year of publishing	
Data	The type of data used in the study (simulated or empirical). In the case of empirical, this column indicates the species studied.	stoch = stochastic simulation, det = deterministic simulation, brl = broilers, pig = pigs
Trait	The trait studied. For simulations, we use arbitrary abbreviations T1-T5, or abbreviations that summarize the $r_{pc}$ and heritability of the trait (e.g., L-M for a trait with low $r_{pc}$ and medium heritability, and M-H for a trait with medium $r_{pc}$ and high heritability).	GLE = gestation length, TNB = total number born, ADG = average daily gain, BF = backfat thickness, LDP = loin depth, LPL = length of productive life, PBA = piglets born alive, LS = litter size, NSB = number stillborn, ADFI = average daily feed intake, BW7 = body weight at 7 d, BW35 = body weight at 35 d
Line	The line for which GEBV were obtained and validated.	LL = Landrace, YY = Yorkshire, DL = Dutch Landrace, LW = Large White
Model	The model used to estimate GEBV	A = additive model, A-D = additive + dominance model, A-DI = additive + dominance + imprinting model, A-BOA = BOA model, A-NOOP = additive model without own performance records, SS-A = single-step additive model, MS = marker selection, MAS = marker assisted selection
$r_{pc}$	The $r_{pc}$ of the trait, estimated from the available data.	
N_PB_ and N_CB_	The number of PB (N_PB_) and CB (N_CB_) animals in the reference population.	
p_geno_	How many CB animals with phenotypes are genotyped, expressed as a fraction of the number of CB animals with phenotypes in the base scenario (N_CB_).	Example: In the base scenario, 500 CB animals are phenotyped. In the alternative scenario, 750 CB animals are phenotyped and genotyped. p_geno_ is then equal to 1.50.
p_PB_ and p_CB_	How many PB and CB reference animals (that have phenotypes) are genotyped, expressed as fractions of the number of PB animals genotyped in the base scenario (N_PB_). Notation: fraction PB; fraction CB	Example: In the base scenario, only 500 PB animals are genotyped. In the alternative scenario, 250 CB genotypes are added and the number of PB genotypes stays the same. p_PB_;p_CB_ is then equal to 1;0.5.
a_PB_ and a_CB_	The relationship between the PB (a_PB_) or CB (a_CB_) animals in the reference population and animals in the validation population.	var = variable (training was done once for subsequent generations), min = minimized using k-means clustering to create training and validation sets
${\;\Delta }_{\rho }$	The difference in accuracy of GEBV between the strategy of interest and the base strategy ($\rho_{1}-\rho_{2}$)	
${\;\Delta }_{R}$	The difference in response to selection per generation between the strategy of interest and the base strategy $\left(R_{1}-R_{2}\right)$, where $R_{1}$ and $R_{2}$ are responses per generation, expressed in additive genetic standard deviations	

### Baseline strategy

The baseline strategy is an additive genomic prediction model and a PB reference population of the same line as the selection candidates, raised in the PB environment. Effectively, this strategy results in estimated average effects for PB performance (${\hat{\alpha }}_{PB}$), and GEBV for PB performance (${\hat{u}}_{PB}$).

## Using Only Purebred Data

In this section, we compare strategies that use a PB reference population. We compare the baseline strategy with strategies that 1) account for dominance in the genomic prediction model, or 2) collect phenotypes of PB raised in a CB environment.

### Accounting for dominance with PB data

This section discusses the benefit of accounting for dominance in the genomic prediction model when a PB reference population is used. Values of $r_{pc}$ lower than one can be a result of interactions between alleles (i.e., non-additive effects) in combination with differences in allele frequencies between parental lines, a phenomenon known as G × G interactions (Wei et al., 1991; Baumung et al., 1997; Duenk et al., 2021). For a locus with only an additive and dominance effect, the average effect depends on the allele frequency “in the mates.” Hence, in PB line 1, the average effect for PB performance depends on the allele frequency in line 1, whereas the average effect for CB performance (i.e., when line 1 is mated to line 2), depends on the allele frequency in line 2 (e.g., Pirchner and Mergl, 1977; Dekkers, 1999). This results in


$$\begin{array}{l} \alpha_{PB}=a+\left(1-2p_{1}\right)d \end{array}$$ (3)

$$\begin{array}{l} \alpha_{CB}=a+\left(1-2p_{2}\right)d, \end{array}$$ (4)

where a is the additive effect, d is the dominance effect, $p_{1}$ is the allele frequency in line 1, and $p_{2}$ is the allele frequency in line 2 (Falconer and Mackay, 1996). Equation (4) suggests that estimates of $\alpha_{CB}$ at markers can be obtained by estimating additive (a) and dominance (d) effects at markers separately, and use the observed marker allele frequency in line 2 ($p_{2}$) to compute $\alpha_{CB}$. This approach allows selecting PB selection candidates for CB performance when there is dominance, and is hereafter called the “dominance model.”

We found three studies that investigated the benefit of using the dominance model instead of the additive model for accuracy and response to selection. Esfandyari et al. (2015b) simulated five generations of selection in two PB parental lines of pigs, where the selected traits had an $r_{pc}$ lower than one only due to dominance. In each generation, the selected sires from the first line were mated to the selected dams from the second line to create crossbreds. In the first generation, a reference population of 1,000 PB animals from each parental line was used to estimate $\alpha_{PB}$ at each marker with an additive model, or to estimate a and d and subsequently $\alpha_{CB}$ with a dominance model. The results showed that the dominance model improved response per generation by 0.07 additive genetic SD (gSD) for an $r_{pc}$ of 0.66, and by 0.04 gSD for an $r_{pc}$ of 0.70 (Table 2).

**Table 2.** Change in accuracy (${\;\Delta }_{\rho }$) and response to selection in CB performance (${\;\Delta }_{R}$) when a dominance model is used instead of an additive model, and only PB phenotypes and genotypes are in the reference population^1^

Study	Data	Trait	Line	N markers	r_pc_	N_PB_	${\;\Delta }_{\rho }$	${\;\Delta }_{R}$
Esfandyari 2015b	stoch	T1	PB line	1,000	0.66	1,000		0.07
Esfandyari 2015b	stoch	T2	PB line	1,000	0.70	1,000		0.04
Esfandyari 2016	pig	LS	LL sires	34,216		2,085	0.03	
Esfandyari 2016	pig	LS	YY sires	35,135		2,145	0.04	
Esfandyari 2018	stoch	T1-G40	PB sire line	4,000	0.82	1,000		*-0.01*
Esfandyari 2018	stoch	T1-G5	PB sire line	4,000	0.82	1,000	0.00	0.01
Esfandyari 2018	stoch	T2-G40	PB sire line	4,000	0.82	1,000		*-0.01*
Esfandyari 2018	stoch	T2-G5	PB sire line	4,000	0.82	1,000	*-0.01*	0.00

^1^Negative values are presented in italics.

In a similar simulation study by Esfandyari et al. (2018), where genetic effects were re-estimated every generation and the average $r_{pc}$ across traits was 0.82, the benefit of the dominance model almost disappeared, with a maximum increase of 0.01 gSD in response to selection per generation (Table 2). Across 40 generations of selection, the dominance model led to a “reduction” of 0.01 gSD in mean response to selection per generation compared with the additive model. The authors concluded that the dominance model was beneficial for response in the short term, but not in the long term. They argued that the additive and dominance model led to different selection responses on the short- and long term because the models result in improvements of different components of CB performance. With the dominance model, CB performance was primarily improved by increasing heterozygosity at overdominant loci (leading to increased heterosis), while with the additive model, CB performance was primarily improved by increasing the performance in the parental lines. Note, however, that these simulations only considered dominance effects but neither epistatic effects nor G × E.

Finally, in an empirical study of litter size in pigs, Esfandyari et al. (2016) compared the accuracy of an additive model with that of a dominance model, using a reference population of about 2,000 animals per line. Their results showed that the dominance model improved $\rho_{CB}$ by 0.03 to 0.04 compared with the additive model. The study did not report estimated $r_{pc}$, but they did mention that dominance genetic variance accounted for about 1% of the phenotypic variance, while additive genetic variance accounted for about 5%.

In summary, the results from these studies suggest that, when only dominance is present, $\rho_{CB}$ and short-term response to selection in CB performance may be improved by using a dominance instead of an additive genomic prediction model, while benefits for long-term response require further study.

### Testing PB animals in a crossbred environment

In this section, we will discuss the benefit of testing PB animals in a CB environment. In addition to G × G interactions, $r_{pc}$ values can be lower than one due to G × E interaction (Falconer, 1952; Lutaaya et al., 2001). When using only PB data, breeders can account for the G × E component of $r_{pc}$ by testing part of the PB animals in a commercial instead of a nucleus environment, and using these animals in the reference population. Because of biosecurity reasons, the PB animals that are tested in a commercial environment cannot be used as selection candidates anymore. Thus, at the same total testing capacity, testing part of the PB in the commercial environment decreases the selection intensity and potentially the response to selection. Hence, testing a proportion of PB animals in a commercial environment results in a trade-off between the advantage of accounting for G × E and the disadvantage of reduced selection intensity.

With simulations of a broiler breeding program, Chu et al. (2018) investigated whether testing part of the PB animals in a commercial environment (C) improves accuracy and response to selection compared with testing all PB animals in the nucleus environment. They considered different $r_{pc}$’s (by simulating G × E interaction only), heritabilities for the trait in C, and proportions of purebreds tested in C. Their results showed that the optimal proportion of PB animals tested in C was 30% (i.e., 896 PB tested in PB environment and 384 PB tested in C). With this scenario, the accuracy of GEBV increased by 0.07 when $r_{pc}$ was 0.9, and by 0.41, when $r_{pc}$ was 0.5 (Table 3). Furthermore, response to selection in CB performance increased by 0.01 gSD when $r_{pc}$ was 0.9, and by 0.09 gSD when $r_{pc}$ was 0.5. These benefits of testing purebreds in C decreased when the heritability for the trait in C decreased, relative to the heritability in the nucleus environment (which was fixed at 0.28). Similar results were found in a simulation study on rainbow trout (Chu et al., 2020), where testing 20% of the PB animals in C increased accuracy by 0.29 when $r_{pc}$ was 0.5, and by 0.14 when $r_{pc}$ was 0.8. As a result, response in CB performance increased by 0.09 gSD when $r_{pc}$ was 0.5, and by 0.02 gSD when $r_{pc}$ was 0.8. The response to selection decreases as the fraction tested in C increased to 40% or 60%, as a consequence of decreased selection intensity. It should be noted that the number of available records per generation in these studies was 1,280 or 1,000, and that with a larger number of records, the optimal proportion tested in C could be smaller (see Discussion).

**Table 3.** Change in accuracy (${\;\Delta }_{\rho }$) and response to selection in CB performance ($\Delta_{R}$) when a fraction of the PB animals were tested in a commercial environment in each generation, compared with when all PB animals were tested in the nucleus environment. (The full version of this table with all comparisons is presented in Supplementary Table S1)^1^

Study	Data	Line	Model	h^2^	r_pc_	N_PB_^2^	% tested in C	a_PB_	a_CB_	${\;\Delta }_{\rho }$	${\;\Delta }_{R}$
Chu 2018	Broilers	L-H	PB sire line	SS-A	0.5	1280	30	0–1	0–0.5		0.10
Chu 2018	Broilers	L-L	PB sire line	SS-A	0.5	1280	30	0–1	0–0.5		0.08
Chu 2018	Broilers	L-M	PB sire line	SS-A	0.5	1280	30	0–1	0–0.5	0.41	0.09
Chu 2018	Broilers	H-H	PB sire line	SS-A	0.9	1280	30	0–1	0–0.5		0.01
Chu 2018	Broilers	H-L	PB sire line	SS-A	0.9	1280	30	0–1	0–0.5		0.00
Chu 2018	Broilers	H-M	PB sire line	SS-A	0.9	1280	30	0–1	0–0.5	0.07	0.01
Chu 2020	Trout	T1	PB line B	SS-A	0.5	1000	20	0–0.125	0–0.125	0.29	0.09
Chu 2020	Trout	T1	PB line B	SS-A	0.5	1000	60	0–0.125	0–0.125	0.44	0.06
Chu 2020	Trout	T2	PB line B	SS-A	0.8	1000	20	0–0.125	0–0.125	0.14	0.02
Chu 2020	Trout	T2	PB line B	SS-A	0.8	1000	60	0–0.125	0–0.125	0.26	0.00

^1^Accuracies were only reported for scenarios with a heritability for CB performance of 0.25.

^2^N_PB_ denotes the number of PB selection candidates that were phenotyped and genotyped in each generation.

In conclusion, when there is G × E interaction, testing a fraction of the PB animals in a commercial environment is beneficial for genomic prediction accuracy, which is only partly translated in additional response to selection due to reduced selection intensity.

## Using Crossbred Data

In this section, we will focus on the benefit of 1) phenotyping, 2) genotyping, or 3) both phenotyping and genotyping CB animals, compared with using PB data only. For each of these strategies, the CB phenotypes and genotypes can either replace PB data in the reference population, or be added to the reference population. In this section, for scenarios that use both PB and CB data, PB and CB performance were modeled as separate, but correlated traits (i.e., genetic effects for PB and CB performance are different and correlated).

When CB data are collected, the genomic prediction model can be refined by either 1) accounting for dominance, 2) considering the breed-origin of alleles (BOA), or 3) allow for correlated effects of alleles between parental lines when data from more than one parental line is used. In the following, we will first discuss the benefit of using CB data while using an ordinary additive genomic prediction model, after which we will discuss the benefits of model improvements.

### Phenotyping crossbreds

In this section, we discuss the benefit of phenotyping CB instead of PB animals, without collecting their genotypes. It is possible to use these phenotypes for genomic prediction when the CB animals have known pedigree links to genotyped PB selection candidates. There are two alternative approaches for this strategy. The first approach is to use CB phenotypes to estimate pedigree-based deregressed EBV (DEBV) of PB relatives. Then, the DEBV and genotypes of these PB relatives can be used to train the genomic prediction model. The second approach is to compute genotype probabilities of the CB animals with phenotypes, based on the genotypes of their parents. The genotype probabilities and phenotypes can be subsequently used to train the genomic prediction model. This approach is comparable to the use of single-step GBLUP, which facilitates the use of phenotypes of ungenotyped animals in the reference population by combining the pedigree-based relationship matrix with the genotype-based relationship matrix (Christensen et al., 2014; Legarra et al., 2014). In pigs and poultry, this second approach may be preferred, because accurate DEBV for CB performance of PB selection candidates are usually not available at the time of selection. Note that the strategies compared in this section either used PB or CB phenotypes, but never both.

The benefit of only phenotyping CB animals was studied by Esfandyari et al. (2015a), who simulated a crossbreeding program of pigs where the selection criterium was a trait with an $r_{pc}$ of 0.78. They compared using a reference population of 2,000 PB phenotypes and genotypes with using a reference population of 2,000 CB phenotypes and genotype probabilities. It is important to note that the animals in the PB reference population had stronger relationships with the selection candidates than the CB animals in the reference population (one versus three generations separated). This difference in relationship between scenarios resembles a scenario where selection decisions are made early in life, so that phenotypes of half-sibs and full-sibs are unavailable. Their simulations included strong dominance effects, and GEBV of PB selection candidates were estimated using a dominance model. The results showed that using 2,000 phenotypes of CB animals and their genotype probabilities improved response to selection by about 0.03 gSD, compared with when 2,000 PB phenotypes and genotypes were used (Table 4). When they also accounted for breed-specific effects of alleles, response to selection improved by 0.06 gSD (see also section on Considering the breed-origin of alleles in crossbreds).

**Table 4.** Change in accuracy (${\;\Delta }_{\rho }$) and response to selection in CB performance ($\Delta_{R}$) when PB phenotypes are replaced with CB phenotypes, when only PB genotypes are available (The full version of this table with all comparisons is presented in Supplementary Table S2)^1^

Study	Data	Trait	Line	Model	r_pc_	N_PB_	p_PB;_ p_CB_	a_PB_	a_CB_	${\;\Delta }_{\rho }$	${\;\Delta }_{R}$
Esfandyari 2015a	stoch	T1	PB line	A-D	0.78	2000	0;1	0–0.5	0–0.125		0.03
Esfandyari 2015a	stoch	T1	PB line	A-D-BOA	0.78	2000	0;1	0–0.5	0–0.125		0.06
See 2020	stoch	T1	PB sire line	SS-A	0.3	2100	0;0.76	0–1	0–0.25	0.09	0.29
See 2020	stoch	T2	PB sire line	SS-A	0.7	2100	0;0.76	0–1	0–0.25	0.01	0.11
See 2020	stoch	T3	PB sire line	SS-A	0.9	2100	0;0.76	0–1	0–0.25	*-0.13*	*-0.06*
Tusell 2020	pig	ADG	PB sire line	A		5137	0;0.54	0–0.5	0–0.25	0.09	
Tusell 2020	pig	RFI	PB sire line	A		5137	0;0.54	0–0.5	0–0.25	*-0.17*	
Tusell 2020	pig	ADG	PB sire line_young	A		3209	0;0.95	0–0.5	0–0.25	*-0.21*	
Tusell 2020	pig	RFI	PB sire line_young	A		3209	0;0.95	0–0.5	0–0.25	*-0.11*	

^1^Negative values are presented in italics.

In another simulation study of three-way CB pigs, See et al. (2020) compared the use of 2,100 PB phenotypes, with the use of 1,600 CB phenotypes in single-step genetic evaluations. Again, the PB animals with phenotypes had stronger relationships with the selection candidates than the CB animals with phenotypes, because it was assumed that own performance and PB full-sib records were available at the time of selection. Their results showed that, with an $r_{pc}$ of 0.7, using CB instead of PB records increased accuracy in the PB sire line by 0.01 and response to selection by 0.11 gSD per generation. This benefit was larger with an $r_{pc}$ of 0.3 (increase of 0.09 in accuracy and 0.29 gSD in response), and disappeared with an $r_{pc}$ of 0.9 (decrease of 0.13 in accuracy and 0.06 gSD in response).

Finally, in an empirical study of pigs, Tusell et al. (2020) compared the use of 5,137 PB phenotypes and genotypes in the reference population, with the use of DEBV and genotypes of 205 PB sires in the reference population. The DEBV of these sires were computed from phenotypes of their 2,774 CB offspring. Unfortunately, $r_{pc}$ values were not reported in this study. Their results showed that, with an additive genomic prediction model and random cross-validation, replacing PB phenotypes with DEBV based on CB phenotypes improved accuracy by 0.09 for ADG, and decreased accuracy by 0.17 for RFI (Table 4). The benefit of replacing PB with CB phenotypes increased when a subset of SNPs (500–2,000 out of 47,000) and a support vector machine (SVM) were used. With this method, accuracy increased by 0.17 for ADG and by 0.04 for RFI. When validation was performed in the two youngest generations in the data, there was a disadvantage of replacing PB with CB phenotypes (between -0.11 and -0.21 across traits and models), except for ADG and when an SVM was used, which showed an increase in accuracy of 0.07.

The results of these studies suggest that replacing phenotypes of PB with those of CB animals can improve accuracy of GEBV and response to selection in CB performance. This benefit was observed for traits with an $r_{pc}$ lower than 0.9, even when the phenotyped CB animals had weaker relationships with the selection candidates than the phenotyped PB animals.

### Genotyping crossbreds

This section discusses the benefit of genotyping CB animals that have already been phenotyped. When CB phenotypes are collected, breeders can decide to collect their genotypes as well. Collecting genotypes of phenotyped CB animals alleviates both the requirements to record their pedigree, and to phenotype CB animals that are closely related to the selection candidates. We compare scenarios that use only CB, or both PB and CB phenotypes in the reference population.

The effect of genotyping crossbreds “instead” of using their genotype probabilities (i.e., p_geno_ was 1.00) on the response to selection was studied by Esfandyari et al. (2015a). They compared a scenario where training was based on phenotypes and genotype probabilities of 2,000 CB animals, with a scenario where genotypes of these CB animals were collected. Results showed that using CB genotypes improved response to selection in CB performance by 0.04 gSD for a trait with an $r_{pc}$ of 0.78 (Table 5). Similarly, See et al. (2020) compared accuracy of single-step genetic evaluations and response to selection of a scenario where 1,600 CB animals were only phenotyped, with a scenario where these CB animals were genotyped as well. Their results showed that genotyping CB animals increased accuracy by 0.23 with an $r_{pc}$ of 0.3, by 0.17 with an $r_{pc}$ of 0.7, and by 0.16 with an $r_{pc}$ of 0.9. Response to selection increased by 0.06 gSD per generation with an $r_{pc}$ of 0.3, by 0.14 with an $r_{pc}$ of 0.14, and by 0.10 with an $r_{pc}$ of 0.10. It is remarkable that in this study, the differences in response to selection across the three different traits did not correspond to the differences in accuracy, despite the fact that all parameters other than $r_{pc}$ were the same across traits. For example, when comparing traits with $r_{pc}=0.3$ and $r_{pc}=0.7$, the difference in accuracy becomes larger, whereas the difference in response becomes smaller. The reasons for this discrepancy are unclear.

**Table 5.** Change in accuracy (${\;\Delta }_{\rho }$) and response to selection in CB performance ($\Delta_{R}$) when CB genotypes are added, for the scenario where only CB phenotypes are used (The full version of this table with all comparisons is presented in Supplementary Table S3)

Study	sp	Trait	Line	Model	r_pc_	N_CB_	p_geno_	a_CB_	${\;\Delta }_{\rho }$	${\;\Delta }_{R}$
Esfandyari 2015a	stoch	T1	PB line	A-D	0.78	2000	1.00	0–0.125		0.04
Esfandyari 2015a	stoch	T1	PB line	A-D-BOA	0.78	2000	1.00	0–0.125		0.04
See 2020	pigs	T1	PB sire line	SS-A	0.3	1600	1.00	0–0.25	0.23	0.06
See 2020	pigs	T3	PB sire line	SS-A	0.9	1600	1.00	0–0.25	0.16	0.10
Sewell 2018	pig	ADG	PB sire line	SS-A		667	1.00	unclear	0.08	
Sewell 2018	pig	ADG	PB sire line	SS-A		667	1.88	unclear	0.31	
Sewell 2018	pig	BF	PB sire line	SS-A		667	1.00	unclear	0.19	
Sewell 2018	pig	BF	PB sire line	SS-A		667	1.88	unclear	0.36	
Sewell 2018	pig	LDP	PB sire line	SS-A		667	1.00	unclear	0.30	
Sewell 2018	pig	LDP	PB sire line	SS-A		667	1.88	unclear	0.57	
Tusell 2020	pigs	ADG	PB sire line_young	A		3059	1.31	0–0.25	0.05	
Tusell 2020	pigs	RFI	PB sire line_young	A		3059	1.31	0–0.25	0.28	

The benefit of genotyping crossbreds for single-step genomic prediction accuracy in pigs was studied by Sewell (2018). When only CB phenotypes (*N* = 1252) and PB genotypes (*N* = 667) were available, genotyping about half of the CB with phenotypes (*N* = 668, p_PB_;p_CB_ was 1;1) improved accuracy by 0.08 for ADG, 0.19 for BF, and by 0.30 for LDP (Table 5). Genotyping all of the crossbreds (*N* = 1252, p_PB_;p_CB_ was 1;1.88) improved accuracy by 0.31 for ADG, 0.36 for BF, and by 0.57 for LDP. The benefit of genotyping CB animals decreased when phenotypes of both PB and CB animals were available: genotyping half of the CB (*N* = 668, p_PB_;p_CB_ was 1;1) in addition to the PB improved accuracy by 0.04 for ADG, 0.03 for BF, and by 0.09 for LDP (Table 6). Genotyping all the CB in that scenario (*N* = 1252, p_PB_;p_CB_ was 1;1.88) led to an increase in accuracy of 0.14 for ADG, 0.06 for BF, and 0.19 for LDP. It is important to note that the accuracy of LDP was strongly negative (-0.27) when none of the CB were genotyped. Unfortunately, an explanation for this negative accuracy (as well as $r_{pc}$ values and relationships between the PB and CB animals) were not reported.

**Table 6.** Change in accuracy (${\;\Delta }_{\rho }$) when CB genotypes are added, for the scenario where both PB and CB phenotypes are used (The full version of this table with all comparisons is presented in Supplementary Table S4)^1^

Study^2^	Trait	Line	Model	r_pc_	N_PB_	p_PB_;p_CB_	a_PB_	a_CB_	${\;\Delta }_{\rho }$	${\;\Delta }_{R}$
Grevenhof 2015	T1	PB line	A	0.7	2000	1;1			0.02	
Grevenhof 2015	T1	PB line	A	0.7	6000	0.5;0.5			0.01	
Grevenhof 2015	T3	PB line	A	0.5	6000	0.5;0.5			0.03	
Grevenhof 2015	T2	PB line	A	0.9	6000	0.5;0.5			*-0.01*	
Grevenhof 2015	LPL	PB line	A-NOOP	0.5	6000	0.5;0.5			0.02	
Grevenhof 2015	LPL	PB line	A-NOOP	0.9	6000	0.5;0.5			*-0.02*	
Grevenhof 2015	T1	PB line	A-NOOP	0.7	2000	1;1			0.08	
Xiang 2017	TNB	LL sires	A-C	0.79	7800	1;0.67	0–1	0.5	0.09	
Xiang 2017	TNB	YY sires	A-C	0.68	7800	1;0.67	0–1	0.5	0.03	
Sewell 2018	ADG	PB sire line	A		667	1;1			0.04	
Sewell 2018	ADG	PB sire line	A		667	1;1.88			0.14	
Sewell 2018	BF	PB sire line	A		667	1;1			0.03	
Sewell 2018	BF	PB sire line	A		667	1;1.88			0.06	
Sewell 2018	LDP	PB sire line	A		667	1;1			0.09	
Sewell 2018	LDP	PB sire line	A		667	1;1.88			0.19	
See 2020	T1	PB sire line	SS-A	0.3	2100	1;0.38	0–1	0–0.25	0.12	0.12
See 2020	T2	PB sire line	SS-A	0.7	2100	1;0.38	0–1	0–0.25	0.08	0.05
See 2020	T3	PB sire line	SS-A	0.9	2100	1;0.38	0–1	0–0.25	0.01	*-0.09*
Sevillano 2018	ADFI	PB sire line	SS-A	0.75–0.88	6594	1;0.45	0.5	0–0.25	0.08	
Sevillano 2018	ADG	PB sire line	SS-A	0.75	6594	1;0.45	0.5	0–0.25	0.09	
Sevillano 2018	BF	PB sire line	SS-A	0.8	6594	1;0.45	0.5	0–0.25	0.04	
Sevillano 2018	LDP	PB sire line	SS-A	0.75–0.88	6594	1;0.45	0.5	0–0.25	0.00	

^1^Negative values are presented in italics.

^2^All studies presented in this table were based on simulations, except for Sevillano 2018, which was on pigs.

Finally, in another study of pigs, Tusell et al. (2020) compared a scenario where 3,059 CB phenotypes were used to estimate DEBV of genotyped PB animals in the reference population, with the use of a reference population of 3,998 CB animals. Their results showed that, using either an additive genomic prediction model or an SVM, genotyping CB animals resulted in a higher accuracy for both ADG (increase of 0.05–0.16), and RFI (increase of 0.11–0.28).

Van Grevenhof and Van Der Werf (2015) used deterministic equations to evaluate the accuracy of a selection index that combines GEBV for CB performance with additional phenotypic information on both PB and CB animals. Their results showed that, with 2,000 PB and 2,000 CB phenotypes for training, genotyping the 2,000 CB “in addition to” the PB improved accuracy by 0.02 for a trait with a heritability of 0.25 and an $r_{pc}$ of 0.7 (Table 6). This benefit was larger (0.04) when the number of genotyped CB animals was doubled to 4,000, or when own performance records of PB selection candidates were unavailable (model A-NOOP, 0.08).

In a scenario with a reference population of 6,000 PB animals, “replacing” half of these records with those of CB animals resulted in a small (0.01) increase in accuracy. For a trait with no own performance records and a lower heritability ($h^{2}=0.12$ for trait LPL; Table 6), this small benefit disappeared. When the entire reference population of 6,000 PB animals was replaced with 6,000 CB animals (p_PB_;p_CB_ was 0;1), the accuracy increased by 0.03. This benefit became larger when $r_{pc}$ was 0.5 (+0.06), or when the size of the PB reference population was a third (p_PB_;p_CB_ was 0;3) of the CB reference population that replaced it (+0.05). With an $r_{pc}$ of 0.9, accuracies tended to decrease (by max. 0.02) when PB genotypes were (partly) replaced by CB genotypes. Note that, for the deterministic prediction of the accuracy, Van Grevenhof and Van Der Werf (2015) assumed that the number of independent chromosome segments that need to be estimated was twice as large in a CB compared with a PB reference population, which is equivalent to assuming that each CB individual was half as informative as a PB individual for selection in a PB line.

The simulation study of See et al. (2020) showed that genotyping CB animals with phenotypes in addition to PB animals with phenotypes increased accuracy and response to selection when $r_{pc}$ was lower than 0.9. For a trait with an $r_{pc}$ of 0.3, accuracy and response to selection (in gSD per generation) both increased by 0.12 when 800 CB animals with phenotypes were genotyped in addition to 2,100 PB reference animals (p_PB_;p_CB_ was 1;0.38). For that trait, genotyping 1,600 CB animals with phenotypes increased accuracy by 0.20 and increased response to selection by 0.17 (Table 6). When $r_{pc}$ was 0.7, the benefit of genotyping CB animals decreased, because accuracy increased by 0.15 and response to selection by 0.10 when 1,600 CB animals were genotyped. For a trait with an $r_{pc}$ of 0.9, accuracy increased by 0.05, but response to selection decreased by 0.07. Again, it is remarkable that in this study, the differences in response to selection across the three different traits did not correspond to the differences in accuracy.

In a dataset of pigs, Xiang et al. (2017) studied the benefit of genotyping 5,200 CB animals with phenotypes while using a single-step genomic prediction model that allows for correlations between genetic effects across the two parental lines and the crossbreds. The number of phenotyped and genotyped PB animals was 7,800 per line. Their results showed that genotyping CB animals increased accuracy of GEBV by 0.09 in line LL where $r_{pc}$ was 0.79, and by 0.03 in line YY where $r_{pc}$ was 0.68 (Table 6). It should be noted that the reported accuracies in this study were derived from the prediction error variance (PEV) of the model, and not with cross-validation.

Finally, Sevillano (2018) evaluated accuracy of GEBV of PB sires in a pig dataset, using a single-step approach. The dataset consisted of about 47,000 PB and 38,000 CB phenotype records. From these animals, about 6,600 PB and 3,000 CB were genotyped. The phenotypes of the 142 youngest sires and their CB offspring were masked, to reflect a scenario where selection decisions are made early in life. Accuracy was evaluated as the correlation between GEBV of these sires and the average performance of their CB offspring. Results showed that including the genotypes of CB animals compared with using only PB genotypes in the training set improved accuracy by 0.04–0.09, for traits with an $r_{pc}$ between 0.75 and 0.88.

In summary, collecting CB genotypes seems to be beneficial for traits with an $r_{pc}$ lower than about 0.9, especially when own phenotypes of PB selection candidates are unavailable. When PB phenotypes are also available, the benefit of adding CB genotypes increases with a decreasing number of PB animals in the reference population.

### Phenotyping and genotyping crossbreds

This section discusses the benefit of phenotyping and genotyping CB animals. In other words, we study the benefit of using a CB reference population. This comparison may be interesting for breeders that do not phenotype CB animals yet, because it directly enables to assess the value of CB information compared with that of PB information for genomic prediction.

Ten studies have investigated the benefit of using CB phenotypes and genotypes, compared with using only PB phenotypes and genotypes. Nine of these studies focused on replacing a PB reference population with a CB reference population (Table 7), while two studies focused on the benefit of adding CB animals to a PB reference population (Table 8). One out of these 10 studies was based on deterministic equations, six were based on simulations, and three were based on empirical data.

**Table 7.** Change in accuracy (${\;\Delta }_{\rho }$) and response to selection in CB performance ($\Delta_{R}$) when PB phenotypes and genotypes are replaced by CB phenotypes and genotypes (i.e., replacing a PB reference population with a CB reference population) (The full version of this table with all comparisons is presented in Supplementary Table S5)^1^

Study	Data	Trait	Line	Model	r_pc_	N_PB_	N_CB_	a_PB_	a_CB_	${\;\Delta }_{\rho }$	${\;\Delta }_{R}$
Dekkers 2007	det	T1		MAS	0.7					0	0.02
Dekkers 2007	det	T3		MAS	0.7					0	0.30
Dekkers 2007	det	T1		MS	0.7					0	0.07
Dekkers 2007	det	T3		MS	0.7					0	0.31
Ibanez-Escriche 2009	stoch	T1	3w dam	A	1	4000	4000	0	0	*-0.09*	
Ibanez-Escriche 2009	stoch	T1	3w sire	A	1	4000	4000	0	0	*-0.11*	
Ibanez-Escriche 2009	stoch	T1	4w PB	A	1	4000	4000	0	0	*-0.09*	
Ibanez-Escriche 2009	stoch	T1	4w PB UR^2^	A	1	4000	4000	0	0	*-0.22*	
Kinghorn 2010	stoch	T1		A		400	400				0.07
Esfandyari 2015a	stoch	T1	PB line	A-D	0.78	2000	2000	0–0.5	0–0.125	0.12	0.08
Hidalgo 2016	pig	GLE	DL line	A	0.94	550	550	0.04	0.03	*-0.14*	
Hidalgo 2016	pig	GLE	LW line	A	0.94	550	550	0.04	0.03	*-0.03*	
Hidalgo 2016	pig	TNB	DL line	A	0.9	914	914	0.04	0.03	*-0.15*	
Hidalgo 2016	pig	TNB	LW line	A	0.9	914	914	0.04	0.03	*-0.18*	
Esfandyari 2018	stoch	T1-G40	PB sire line	A	0.82	2000	2000	0–0.5	0–0.25		0.02
Esfandyari 2018	stoch	T1-G5	PB sire line	A	0.82	2000	2000	0–0.5	0–0.25	0.07	0.09
Duenk 2019	broilers	BW35	PB sire line	A	0.96	4471	4445	0–0.125	0–0.125	*-0.10*	
Duenk 2019	broilers	BW7	PB sire line	A	0.8	4687	4655	0–0.125	0–0.125	0.00	
See 2020	stoch	T1	PB sire line	SS-A	0.3	2100	1600	0–1	0–0.25	0.32	0.34
See 2020	stoch	T3	PB sire line	SS-A	0.9	2100	1600	0–1	0–0.25	0.03	0.04
Tusell 2020	pigs	ADG	PB sire line	A		3209	3998			0.10	
Tusell 2020	pigs	RFI	PB sire line	A		3209	3998			0.04	
Tusell 2020	pigs	ADG	PB sire line_young	A		3209	3998			*-0.16*	
Tusell 2020	pigs	RFI	PB sire line_young	A		3209	3998			0.17	

^1^Negative values are presented in italics.

^2^UR = unrelated parental lines.

**Table 8.** Change in accuracy (${\;\Delta }_{\rho }$) and response to selection in CB performance ($\Delta_{R}$) when CB phenotypes and genotypes are added to a PB reference population (The full version of this table with all comparisons is presented in Supplementary Table S6)

Study	Data	Trait	Line	Model	r_pc_	N_PB_	p_PB_;p_CB_	a_PB_	a_CB_	${\;\Delta }_{\rho }$	${\;\Delta }_{R}$
Gonzalez-Dieguez 2020	stoch	T1	PB line	A-D	0.46	2032	1;1	0–0.5	0–0.125		0.17
Gonzalez-Dieguez 2020	stoch	T2	PB line	A-D	0.3	2032	1;1	0–0.5	0–0.125		0.37
Gonzalez-Dieguez 2020	stoch	T3	PB line	A-D	0.42	2032	1;1	0–0.5	0–0.125		0.30
Gonzalez-Dieguez 2020	stoch	T4	PB line	A-D	0.68	2032	1;1	0–0.5	0–0.125		0.18
See 2020	stoch	T1	PB line	SS-A	0.3	2100	1;0.38	0–1	0–0.25	0.26	0.39
See 2020	stoch	T1	PB line	SS-A	0.3	2100	1;0.76	0–1	0–0.25	0.34	0.44
See 2020	stoch	T3	PB line	SS-A	0.9	2100	1;0.38	0–1	0–0.25	0.05	0.25
See 2020	stoch	T3	PB line	SS-A	0.9	2100	1;0.76	0–1	0–0.25	0.10	0.28

A simulation study (Ibañez-Escriche et al., 2009) of three- and four-way crossbreeding programs illustrated that, when $r_{pc}$ is equal to one, a CB reference population results in a 0.09 to 0.11 lower accuracy than an equally sized PB reference population. When parental lines were completely unrelated, the disadvantage of a CB reference population was even larger (0.22 lower accuracy than with a PB reference population), suggesting that there is a disadvantage of using a CB reference population when $r_{pc}$ is one. The authors suggested that this disadvantage was due to the difference in LD between the PB selection candidates and the CB reference population, which was supported by the observation that this disadvantage became smaller when parental lines were more related, or when marker density was increased. This result undoubtedly depends on the genetic architecture of the trait that is simulated. For example, the disadvantage of using a CB reference population due to differences in LD may be smaller when the number of QTL affecting the trait is larger.

The results of the deterministic study showed that, for a trait with an $r_{pc}$ of 0.7, response to selection was between 0.02 and 0.31 gSD higher with a CB reference population than with a PB reference population (Dekkers, 2007). This difference in response depended on the accuracy of genomic prediction that was used in the equations. In line with this result, a simulation study showed that, in the presence of dominance, response to selection per generation was increased by 0.07 gSD with a reference population of 400 CB animals, compared with a PB reference population of the same size ($r_{pc}$ not reported) (Kinghorn et al., 2010). Another simulation study showed that for a trait with an $r_{pc}$ of 0.78, response to selection in CB performance improved by 0.08 and genomic prediction accuracy by 0.12 gSD with a CB reference population compared with with a PB reference population while using a dominance model (Esfandyari et al., 2015a). In a similar study where $r_{pc}$ was somewhat higher (0.82), genomic prediction accuracy improved by 0.07 when a CB reference population was used. Response to selection on the short term (across the first five generations) increased by 0.09 gSD per generation, whereas response to selection on the long term (across the first 40 generations) increased by 0.02 gSD per generation (Esfandyari et al., 2018). Note that Esfandyari et al. (2015a, 2018) simulated a trait influenced only by additive and dominance gene action, and did not consider epistasis or G × E interaction. Finally, See et al. (2020) studied the benefit of using 1,600 CB animals in the reference population instead of 2,100 PB animals, for traits with different simulated $r_{pc}$. When $r_{pc}$ was 0.3, accuracy increased by 0.32 and response to selection by 0.34 gSD per generation. Traits with a lower $r_{pc}$ showed a smaller benefit of using a CB reference population, with an increase of 0.18 in accuracy and 0.25 gSD in response when $r_{pc}$ was 0.7, and an increase of 0.03 in accuracy and 0.04 gSD in response when $r_{pc}$ was 0.9. In summary, results from simulation studies consistently indicate that a CB reference population can be beneficial for accuracy and response to selection, at least when $r_{pc}$ is lower than about 0.9.

In contrast to the consistent results found with simulations, results from empirical studies vary. In data of pigs, Hidalgo et al. (2016) found that accuracy was 0.03–0.14 lower with a CB reference population compared with a PB reference population, for a trait with an $r_{pc}$ of 0.94. Surprisingly, this disadvantage of a CB reference population was even larger (0.15–0.18 lower) for a trait with a lower $r_{pc}$ of 0.9. The authors suggested that the lower accuracy with a CB reference population was due to weaker relationships between the CB animals and validation population, compared with the PB animals and validation population. In addition, the number of animals used in this study was quite limited (max. 914 PB and CB genotypes). In real data of broiler chicken, Duenk et al. (2019b) compared genomic prediction accuracy when using about 4,500 PB or CB animals in the reference population. Their results showed that the accuracy of genomic prediction was 0.10 lower with a CB compared with a PB reference population for a trait with an $r_{pc}$ of 0.96, and that they were equal for a trait with an $r_{pc}$ of 0.80. Finally, Tusell et al. (2020) studied the difference in accuracy between strategies that either used a reference population of 3,209 PB or 3,998 CB pigs. GEBV were estimated with either an additive genomic prediction model or a support vector machine (SVM), and accuracies were obtained with either random cross-validation or validation in the two youngest generations. With random cross-validation, the accuracy was higher with a CB compared with a PB reference population, both for average daily gain (ADG, increase of 0.10–0.15) and residual feed intake (RFI, increase of 0.04–0.08). With validation in the youngest generations, a CB reference population was beneficial for ADG when using an additive model (increase of 0.17), and for RFI when using a SVM (increase of 0.23). However, there was a disadvantage of using a CB reference population for ADG when using an SVM (decrease of 0.16), and for RFI when using an additive model (decrease of 0.05). The authors note, however, that the accuracies obtained with validation in the youngest two generations should be interpreted with caution, because they were obtained with only a single validation split.

The benefit of adding CB information to a PB reference population, instead of replacing the PB information, was studied by González-Diéguez et al. (2020). They simulated $r_{pc}$ values lower than one by including both G × E interaction and dominance effects, and studied the effects of adding 2,000 CB animals to a PB reference population of 2,000 animals, for selection response over 10 generations. With a PB reference population, GEBVs were computed using an additive model, whereas with a combined PB and CB reference population, GEBVs were computed using a dominance model. Results showed that when $r_{pc}$ was 0.46 and heritability was 0.10, including CB animals to the reference population increased response to selection by 0.17 gSD per generation (Table 8). With larger (and likely unrealistic) dominance effects, $r_{pc}$ dropped to 0.30, and the benefit of including CB information increased to 0.37. This benefit decreased to 0.30 when the narrow-sense heritability was increased from 0.10 to 0.30, resulting in an $r_{pc}$ of 0.42. Finally, when the effect of G × E was small, the $r_{pc}$ was equal to 0.68, and including CB information increased response by 0.18.

In agreement with these results, the simulation study of See et al. (2020) showed that adding CB information to a PB reference population increased accuracy and response to selection, regardless of $r_{pc}$. For a trait with an $r_{pc}$ of 0.3, accuracy increased by 0.26 and response to selection increased by 0.39 when 800 CB animals were added to a reference population of 2,100 PB animals (p_PB_;p_CB_ was 1;0.38). For that trait, adding 1,600 CB animals increased accuracy by 0.34 and increased response to selection by 0.44 (Table 8). When $r_{pc}$ was 0.7, the benefit of adding CB information decreased, because accuracy increased by 0.22 and response to selection by 0.29 when 1,600 CB animals were added. For a trait with an $r_{pc}$ of 0.9, accuracy increased by 0.10, and response to selection increased by 0.28.

In summary, it seems that replacing a PB with a CB reference population is beneficial in terms of accuracy and response to selection, for traits with an $r_{pc}$ lower than about 0.80, but only when the PB and CB reference populations are of similar size, and when the PB and CB animals in the reference population are equally related to the selection candidates. The benefits of adding CB information to a PB reference population has not been studied extensively, but results from two simulation studies suggest that this strategy can greatly improve response to selection, at least for traits with an $r_{pc}$ lower than 0.9.

#### Accounting for dominance with CB data

In this section, we will focus on the benefit of using a dominance model compared with using an additive model when CB phenotypes and genotypes are available. Similar to with a PB reference population (see section “Accounting for dominance with PB data”), a CB reference population can be used to estimate a and d at all markers with the dominance model, and average effects for CB performance in parental lines can be computed using appropriate allele frequencies (see equation (4)).

Zeng et al. (2013) studied the benefit of using a dominance model over using an additive model in simulations of a CB breeding program. They simulated two traits where dominance variance was either 17% or 10% of the phenotypic variance, while allowing for overdominance. Note that for both these traits, the amount of dominance variance was larger than reported in empirical studies (e.g., Guo et al., 2016; Vitezica et al., 2018; González-Diéguez et al., 2019), and that the simulations did not include epistasis or G × E interaction. Their results showed that the use of a dominance model increased the response across 20 generations of selection by 0.02 gSD per generation with 17% dominance variance, and by 0.01 with 10% dominance variance (Table 9). In addition, the dominance model resulted in a reduction in response to selection (-0.01) for a trait with no dominance variance at all, suggesting that increased model complexity had limited negative consequences for selection response (Zeng et al., 2013).

**Table 9.** Change in accuracy (${\;\Delta }_{\rho }$) and response to selection in CB performance ($\Delta_{R}$) when a dominance model is used instead of an additive model, and CB phenotypes and genotypes are in the reference population^1^

Study	Data	Trait	Line	Model	N markers	r_pc_	N_PB_	N_CB_	${\;\Delta }_{\rho }$	${\;\Delta }_{R}$
Zeng 2013	stoch	Large-D		A-D	10000			1000		0.02
Zeng 2013	stoch	Real-D		A-D	10000			1000		0.01
Zeng 2013	stoch	No-D		A-D	10000			1000		*-0.01*
Xiang 2016a	pigs	TNB	CB TGV	A-D	41009	0.70	2675	4094	0.00	
Xiang 2016a	pigs	TNB	CB TGV	A-DI	41009	0.70	2675	4094	0.00	
Christensen 2019	pigs	ADG	sires	A-D/A-DI	6316	0.75	2595	2426	0.00	
Christensen 2019	pigs	BF	sires	A-D/A-DI	6316	0.96	2595	2426	0.00	
Christensen 2019	pigs	CONF	sires	A-D/A-DI	6316	0.83	2595	2426	0.00	
Christensen 2019	pigs	FCR	sires	A-D/A-DI	6316	0.87	2595	2426	0.00	

^1^Negative values are presented in italics.

In two empirical studies of pigs, the dominance model resulted in similar accuracy as the additive model. In both these studies, the reference population consisted of both PB and CB animals, and additive and dominance effects were estimated with a model that considered PB and CB performance as different, but genetically correlated traits. First, Xiang et al. (2016a) analyzed total number born (TNB), which had an estimated $r_{pc}$ of 0.70. Note that the accuracies reported in this study were for total genetic values of CB animals, and not breeding values of PB animals. Hence, any observed benefits of the dominance model in this study probably overestimate the benefit for accuracy of GEBV in parental lines. Yet, the results showed that the accuracy of the dominance model was the same as that of the additive model, regardless of whether inbreeding was included in these models or not (Table 9). Similar results were obtained by Christensen et al. (2019), who evaluated accuracy of breeding values for three-way CB performance in a PB sire line; they analyzed four traits, with estimated $r_{pc}$ ranging from 0.75 to 0.96. Their results also showed that the accuracy of the dominance model was the same as that of the additive model, regardless of $r_{pc}$ and whether inbreeding was included or not.

In summary, the results from these empirical studies suggest that when the reference population includes CB animals, the use of a dominance model instead of an additive model did not improve accuracy. In simulations, the use of a dominance model only slightly improved long-term response to selection when dominance is present. This is in contrast with a scenario where the reference population only consists of PB animals, because the dominance model was beneficial in those cases (see section “Accounting for dominance with PB data”). It should be noted, however, that the benefit of the dominance model in a CB reference population was only studied for traits with a relatively high $r_{pc}$.

#### Considering the breed-origin of alleles in crossbreds

This section discusses the benefit of considering the breed-origin of alleles in CB reference animals. Including phenotypes and genotypes of CB animals in the reference population may improve $\rho_{CB}$, because it accounts for the G × E component of $r_{pc}$. With a simple additive model, the effects of marker alleles on the CB phenotype are assumed to be independent of the breed-origin of these alleles. In other words, the effects of genes are “uniquely defined” in the CB population (Stuber and Cockerham, 1966). In reality, however, the effects of marker alleles can be breed-specific because of 1) differences in marker-QTL LD between parental lines (Wientjes et al., 2015), 2) imprinting effects (O’Brien and Wolf, 2019), and 3) dominance (and likely epistasis) in combination with differences in QTL allele frequencies between parental lines (Wei et al., 1991). The latter can be seen from equation (4), which shows that the average effect of an allele that is transmitted from the first parental line to a CB offspring depends on the allele frequency in the second line, and vice versa (e.g., Pirchner and Mergl, 1977; Dekkers, 1999; Duenk, 2020). Hence, with a CB reference population, the additive model may be refined by estimating breed-specific effects of marker alleles in the crossbreds, which requires that the breed-origin of alleles (BOA) in CB animals is known. Such a model was termed “according to origin” by Stuber and Cockerham (1966). Hereafter, we will call a model that considers the breed-origin of alleles in crossbreds the BOA model.

Seven papers have investigated the benefit of BOA models, four of which were based on simulations, and three on real data. In simulations where $r_{pc}$ was equal to one, considering the BOA only increased accuracy when marker density was relatively low, the number of animals in the reference population was relatively high, and the parental breeds were distantly or unrelated (Ibañez-Escriche et al., 2009). For example, when the reference population consisted of 4,000 CB animals and parental breeds were distantly related, considering the BOA increased accuracy by 0.01 in parental lines of a four-way cross, and by 0.02 in a dam line of a three-way cross (Table 10). In the sire line of a three-way cross, considering the BOA was unfavorable, because accuracy decreased by 0.03. The authors suggested that the limited benefit or disadvantage of considering the BOA in these scenarios was partly due to the relatively high marker density used, and the high similarity of marker-QTL LD between parental lines. Indeed, with a very low marker density and unrelated parental lines, the accuracy increased by 0.08 when the BOA was considered (Ibañez-Escriche et al., 2009). It is remarkable, however, that with high marker density and a large reference population, the disadvantage of considering the BOA was usually larger with unrelated parental lines compared with with distantly related lines (e.g. 0.06 vs. 0.08 in a three-way sire line). The authors argued that for unrelated parental lines, many markers segregate in only one of the parental lines, effectively reducing marker density and the advantage of considering the BOA. In addition, models for CB performance that consider the BOA may be at a disadvantage, because these models require the estimation of more effects, compared with models that ignore the BOA. For example, when analyzing performance of three-way CB animals to estimate BV in all parental lines, a model that considers the BOA requires the estimation of three times as many effects as a model that ignores the BOA (Ibañez-Escriche et al., 2009).

**Table 10.** Change in accuracy (${\;\Delta }_{\rho }$) and response to selection in CB performance ($\Delta_{R}$) when the breed-origin of alleles (BOA) in crossbreds is considered, compared with when the BOA is ignored (The full version of this table with all comparisons is presented in Supplementary Table S7)^1^

Study	Data	Trait	Line^2^	Model	r_pc_	N_PB_	N_CB_	${\;\Delta }_{\rho }$	${\;\Delta }_{R}$
Ibanez-Escriche 2009	stoch	T1	3w dam	A-BOA	1		4000	0.02	
Ibanez-Escriche 2009	stoch	T1	3w sire line	A-BOA	1		4000	*-0.03*	
Ibanez-Escriche 2009	stoch	T1	4w PB	A-BOA	1		4000	0.01	
Ibanez-Escriche 2009	stoch	T1	3w sire line	A-BOA	1		4000	*-0.06*	
Kinghorn 2010	stoch	T1		A-BOA			400		0.03
Zeng 2013	stoch	High-D		A-BOA			1000		0.00
Zeng 2013	stoch	Real-D		A-BOA			1000		*-0.01*
Zeng 2013	stoch	No-D		A-BOA			1000		*-0.06*
Esfandyari 2015a^3^	stoch	T1	PB line	A-D-BOA	0.78		2000		0.02
Esfandyari 2015a	stoch	T2	PB line	A-D-BOA	0.78		500		*-0.01*
Esfandyari 2015a	stoch	T3	PB line	A-D-BOA	0.78		8000		0.03
Sevillano 2017	pig	ADG	PB sires	A-BOA	0.52	1900	1200	0.01	
Sevillano 2017	pig	BF	PB sires	A-BOA	0.69	1900	1200	0.00	
Sevillano 2017	pig	LDP	PB sires	A-BOA	0.55	1900	1200	*-0.01*	
Sevillano 2019	pig	ADG	PB LL	A-BOA	0.44	3228	2816	0.01	
Sevillano 2019	pig	ADG	PB sires	A-BOA	0.66	7575	2816	0.00	
Sevillano 2019	pig	ADG	PB LW	A-BOA	0.49	12794	2816	0.01	
Duenk 2019	brl	BW35	PB sires	A-BOA	0.96		4445	*-0.04*	
Duenk 2019	brl	BW7	PB sires	A-BOA	0.8		4655	0.04	

^1^Negative values are presented in italics.

^2^UR = unrelated parental lines.

^3^The model that considers the BOA is written as a model that accounts for imprinting effects. But in these simulations, they are (probably) equivalent (there was no epistasis). However, the BOA model may suffer from reduced power and deviations in genotype probabilities from expectation, while the imprinting model may not.

In another simulation study, Kinghorn et al. (2010) compared response between genomic selection scenarios that either ignored or considered the BOA in CB animals. They simulated dominance effects to ensure that $r_{pc}$ was lower than one, but the exact value of $r_{pc}$ was not reported. An important aspect of this study was that the QTL genotypes and genotypic QTL effects were assumed to be known, so there was no effect of differences in LD between parental lines. Hence, they compared scenarios where GEBV of PB selection candidates were computed from the simulated average effects in the CB animals, either allowing for breed-specific effects of alleles or not (Note that with only dominance and known QTL effects, the use of an additive model that considers the BOA in CB data leads to the same result as the use of a dominance model in PB data.). The authors observed that the response to selection increased by 0.03 when the BOA was considered, compared with when the BOA was ignored (Table 10).

Zeng et al. (2013) studied the benefit of considering the BOA by simulating two parental lines that were separated for 55 generations before crossbreeding started, resulting in differences in LD between parental lines. In addition to differences in LD, they simulated dominance effects to introduce breed-specific marker effects. Their results showed that, without dominance, considering the BOA decreased response to selection by 0.06 gSD, compared with when the BOA was ignored (Table 10). With realistic dominance variance, considering the BOA decreased response to selection by 0.01 gSD per generation, and with large dominance variance, considering the BOA resulted in the same response to selection as ignoring it. They concluded that the benefit of considering the BOA largely depends on the importance of dominance.

It has been argued that a model that considers the BOA may have reduced power to estimate marker effects compared with a model that ignores the BOA (Kinghorn et al., 2010). Any benefit of the BOA model may, therefore, only be seen in large reference populations. The effect of reference population size on the benefit of the BOA model was studied by Esfandyari et al. (2015a). They simulated two parental lines that were separated for 300 generations, and simulated a trait with dominance effects, resulting in an $r_{pc}$ of 0.78. The results showed that considering the BOA led to a smaller response to selection with a reference population of 500 CB animals (-0.01 gSD per generation), and to a larger response to selection with a reference population of 2,000 (+0.02) or 8,000 (+0.03) CB animals (Table 10). In addition, the authors showed that when the parental lines were more closely related (i.e., 200 instead of 400 generations separated), the benefit of considering the BOA with a reference population of 2,000 CB animals disappeared.

The first empirical study on the benefit of BOA models for the accuracy of genomic prediction used a three-way cross of pigs (Sevillano et al., 2017). This study evaluated multivariate models with covariances between genetic effects in each PB line and the crossbreds, but without covariances between the three PB parental lines. The reference populations consisted of 1,750 to 5,000 PB animals and about 1,300 CB animals. For average daily gain (ADG), that had an estimated $r_{pc}$ of 0.30 in dam line LL and of 0.52 in the sire line, considering the BOA increased the accuracy of genomic prediction by 0.06 in the dam line and by 0.01 in the sire line (Table 10). For backfat thickness (BF) and loin depth (LDP), that had a higher estimated $r_{pc}$ ranging from 0.55 to 0.7, considering the BOA did not improve genomic predictions, or led to a decrease in accuracy of 0.01 to 0.02. In a follow-up study on a dataset with about twice as many CB records and 2–4 times as many PB records, the trait ADG had an estimated $r_{pc}$ of 0.44 in dam line LL and of 0.66 in the sire line, and considering the BOA increased accuracy by 0.01 in the dam line, while there was no benefit in the sire line (Sevillano et al., 2019).

Finally, the benefit of considering the BOA in a reference population of about 4,500 CB broilers was studied by Duenk et al. (2019b). Their results showed that considering the BOA decreased accuracy by 0.04 for body weight at 35 d ($r_{pc}=0.96$), but increased accuracy by 0.04 for body weight at 7 d ($r_{pc}=0.8$, Table 10). Although the parental lines were believed to be distantly related, additional analysis revealed that alleles in CB that originated from the F1 dam line had predictive value for GEBV in the sire line, especially for BW35. This last result suggests that although parental lines were distantly related, the apparent effects of marker alleles on the CB phenotype did not strongly depend on breed origin, suggesting that a model that ignores the BOA is more appropriate.

In summary, the theory and above results suggest that considering the BOA can be beneficial when $r_{pc}$ is low (below ~0.8) due to dominance, and with a large CB reference population. At marker densities typically used in livestock (e.g., 50k markers), it seems that the benefit of considering the BOA does not require large differences in LD between parental lines.

### Benefit of collecting CB information while considering the BOA

In this section, we compare strategies that considered the BOA in CB animals, with scenarios that do not collect CB information. In some cases, there may be no benefit of collecting CB information when the BOA is ignored, but there may be a benefit when the BOA is considered. Hence, this comparison may be relevant for breeders that need to decide whether they should collect CB information.

In a simulation study where $r_{pc}$ was equal to one, replacing a PB reference population with a CB reference population while considering the BOA resulted in a decrease of 0.07 – 0.14 in accuracy for distantly related parental breeds (Table 11, Ibañez-Escriche et al., 2009). When parental breeds were unrelated, accuracy even decreased by 0.32. They argued that this reduction in accuracy was probably due to the higher number of effects that need to be estimated with a CB reference population while the BOA is considered, compared with a PB reference population. Furthermore, there was no advantage of using a CB over a PB reference population in this study, because $r_{pc}$ was equal to one. In contrast to these results, the simulation study by Kinghorn et al. (2010) showed that using a CB reference population while considering the BOA led to a considerable increase (+0.10) in response, compared with using a PB reference population. In their simulations, they ensured that $r_{pc}$ was lower than one by simulating dominance effects, but the value of $r_{pc}$ was not reported. Furthermore, they assumed that QTL genotypes and genotypic QTL effects (i.e., a and d) were known, so differences in LD between parental lines did not play a role.

In line with the results from Kinghorn et al. (2010), a more recent simulation study performed by Esfandyari et al. (2015a) showed that using a CB reference population while considering the BOA resulted in 0.10 gSD more response per generation compared with using a PB reference population (Table 11). They simulated a pig breeding program and a trait with an $r_{pc}$ of 0.78 by including dominance effects only, and used reference populations of 2,000 animals. Note that in this simulation study, the CB reference population was less related to the selection candidates than the PB reference population. This difference in relationships is similar to practical situations, because at the time of selection, the available CB animals with phenotypes and genotypes are usually less related to the selection candidates than the PB animals with phenotypes and genotypes.

The effect of differences in relatedness between the CB reference animals and selection candidates for the benefit of collecting CB information was studied by Wientjes et al. (2020), who simulated a pig breeding program. They compared the use of a PB reference population with the use of a CB reference population while considering the BOA, for two-way or four-way crossbreeding, and with or without multiplication steps to produce crossbreds. With an $r_{pc}$ of 0.75 and a heritability for PB and CB performance of 0.20, a reference population of 2,400 two-way CB animals increased accuracy by 0.10, compared with a PB reference population of the same size (Table 11). This benefit increased to 0.16 when the size of the reference populations increased to 9,600. In contrast, with a reference population of four-way CB animals, the accuracy was similar to when a PB reference population was used (difference of -0.01 to 0.01), regardless of the size of the reference population. Furthermore, for both two-way and four-way CB reference populations, the benefit of a CB reference population decreased a little (by about 0.02) if there was a multiplication step in the breeding program (scenarios _1MP in Table 11). These results show that the benefit of a CB reference population over a PB reference population depends on the genetic relatedness of the CB vs. PB reference animals to the selection candidates. Finally, the authors simulated traits with a heritability in CB animals that was much smaller than in PB animals (0.05 vs. 0.20). For those traits, using two-way or four-way CB information resulted in higher accuracies than using PB information when $r_{pc}$ was 0.50, but not when $r_{pc}$ was 0.75 (Wientjes et al., 2020).

**Table 11.** Change in accuracy (${\;\Delta }_{\rho }$) and response to selection in CB performance ($\Delta_{R}$) when the breed-origin of alleles (BOA) in crossbreds is considered, compared with when CB genotypes are not collected (The full version of this table with all comparisons is presented in Supplementary Table S8)^1^

Study	Data	Trait	Line^2^	r_pc_	N_PB_	N_CB_	a_PB_	a_CB_	${\;\Delta }_{\rho }$	${\;\Delta }_{R}$
Ibanez-Escriche 2009	stoch	T1	3w dam	1	4000	4000	0	0	*-0.07*	
Ibanez-Escriche 2009	stoch	T1	3w sire	1	4000	4000	0	0	*-0.14*	
Ibanez-Escriche 2009	stoch	T1	4w PB	1	4000	4000	0	0	*-0.08*	
Ibanez-Escriche 2009	stoch	T1	4w PB UR^1^	1	4000	4000	0	0	*-0.32*	
Kinghorn 2010	stoch	T1			400	400				0.10
Esfandyari 2015a	stoch	T1	PB line	0.78	2000	2000	0–0.5	0–0.125		0.10
Xiang 2016b	pig	TNB	LL sires	0.79	7800	5200	0–1	0.50	0.04	
Xiang 2016b	pig	TNB	YY sires	0.68	7800	5200	0–1	0.50	0.07	
Lopes 2017	pig	GLE-LL	CB	0.9	832	832	0	0	0.23	
Lopes 2017	pig	GLE-LW	CB	0.9	832	832	0	0	0.11	
Lopes 2017	pig	LS-LL	CB	0.9	832	832	0	0	0.16	
Lopes 2017	pig	LS-LW	CB	0.9	832	832	0	0	0.17	
Duenk 2019	broilers	BW35	PB sire line	0.96	4471	4445	0–0.125	0–0.125	*-0.14*	
Duenk 2019	broilers	BW7	PB sire line	0.8	4687	4655	0–0.125	0–0.125	0.04	
Wientjes 2020	pigs	T1	2CB	0.75	2400	2400	0–0.5	0–0.0625	0.10	
Wientjes 2020	pigs	T1	2CB_1MP	0.75	2400	2400	0–0.5	0–0.0156	0.08	
Wientjes 2020	pigs	T1	4CB	0.75	2400	2400	0–0.5	0–0.0156	*-0.01*	
Wientjes 2020	pigs	T2	2CB	0.5	2400	2400	0–0.5	0–0.0625	0.28	
Wientjes 2020	pigs	T2	2CB_1MP	0.5	2400	2400	0–0.5	0–0.0156	0.27	
Wientjes 2020	pigs	T2	4CB	0.5	2400	2400	0–0.5	0–0.0156	0.16	

^1^Negative values are presented in italics.

^2^UR = unrelated parental lines.

Results from empirical studies usually demonstrated a benefit of using a CB reference population while considering the BOA, compared with using a PB reference population. For example, Lopes et al. (2017) found higher accuracies with a CB reference population while the BOA was considered compared with with a PB reference population (increase of 0.11 to 0.23, Table 11). Both the PB and alternative CB reference population consisted of 832 animals, and the estimated $r_{pc}$ was 0.90. In addition, Duenk et al. (2019b) found that considering the BOA in a CB reference population resulted in a 0.03 higher accuracy than with a PB reference population when $r_{pc}$ was 0.80. When $r_{pc}$ was 0.96, however, it was more beneficial to use a PB reference population (difference of 0.14). Finally, in a study on pigs, Xiang et al. (2016b) investigated the benefit of genotyping CB animals that already have phenotypes. They considered a single-step scenario where about 7,800 PB phenotypes (per line) and 5,200 CB phenotypes were available, and only the PB animals were genotyped. The CB animals with phenotypes were mostly offspring of the genotyped PB animals in the reference population. Results showed that genotyping the CB and considering the BOA increased the PEV-based accuracy of GEBV (of genotyped selection candidates) by 0.07 when $r_{pc}$ was 0.68, and by 0.04 when $r_{pc}$ was 0.79 (Table 11). Note, however, that these improvements in accuracy were similar to those resulting from genotyping crossbreds while allowing for correlated effects between parental lines (i.e., Xiang et al. (2017) in “Genotyping crossbreds”), suggesting that the observed benefit did not come from considering the BOA.

In conclusion, the results suggest that using CB information while considering the BOA in crossbreds can be beneficial for traits with an $r_{pc}$ lower than about 0.9, with comparable heritabilities for PB and CB performance, and the CB reference animals are sufficiently related to the selection candidates. When $r_{pc}$ is close to one, the heritability for CB performance is much lower than for PB performance, or the CB reference animals are weakly related to the selection candidates, using CB information while considering the BOA may not be beneficial for accuracy and selection response.

### Allowing for correlated effects across parental lines and crossbreds

This section discusses the benefit of allowing for correlated genetic effects between parental lines. In the aforementioned scenarios where both PB and CB data were used, GEBV were usually computed from a multivariate model, assuming different and correlated genetic effects between each parental line and the crossbreds, but uncorrelated genetic effects between parental lines. Moreover, uncorrelated genetic effects between parental lines are assumed when the BOA in crossbreds is considered. However, genetic effects between PB lines can be correlated in reality, which is reflected by non-zero values of genetic correlations between populations as observed in the literature. The genomic prediction model may, therefore, be improved by allowing for correlated genetic effects across all parental lines and the crossbreds. This improvement leads to a family of models where genetic effects are uniquely defined and correlated, which has been developed and studied by Christensen et al. (2015), Vitezica et al. (2016), and Xiang et al. (2017). Hereafter, we will call this model the “correlated effects” model. The benefit of this model is that information can be shared between all PB parental lines and the crossbreds, possibly increasing accuracy of GEBV for CB performance.

As mentioned in the previous section, Xiang et al. (2016b) analyzed data from 7,800 PB (per line) and 5,200 CB pigs using a single-step model that assumed different and correlated genetic effects between PB and CB performance, but uncorrelated genetic effects between parental lines. In scenarios where CB animals were genotyped, they considered the BOA. In another study, Xiang et al. (2017) used the correlated effects model to analyze the same data, and, therefore, we were able to compare accuracies across these two studies. This comparison showed that allowing for correlated effects across parental lines increased accuracy by 0.03 to 0.08 for a trait with an $r_{pc}$ of 0.68 for line YY and 0.79 for line LL (Table 12). This result suggests that the correlated effects model may outperform a model where genetic effects are assumed to be uncorrelated across parental lines. Note that Xiang et al. (2016b, 2017) determined accuracy using the PEV of the model, instead of using validation.

**Table 12.** Difference in accuracy (${\;\Delta }_{\rho }$) between a model that allows for covariance between genetic effects for PB performance between parental lines, and a model that does not model covariance between parental lines

Paper	Data	Trait	Line	$r_{pc}$	N_PB_	N_CB_	CB geno	${\;\Delta }_{\rho }$
Xiang 2016b, 2017	pig	TNB	LL sires	0.79	7800	5200	no	0.03
Xiang 2016b, 2017	pig	TNB	LL sires	0.79	7800	5200	yes	0.07
Xiang 2016b, 2017	pig	TNB	YY sires	0.68	7800	5200	no	0.08
Xiang 2016b, 2017	pig	TNB	YY sires	0.68	7800	5200	yes	0.03

## Discussion

We compared genomic prediction strategies for estimating BV for CB performance of PB selection candidates that differed in 1) the genomic prediction model that was applied, or 2) the data that was used in the reference population. For each strategy, we have summarized its strengths and weaknesses in Table 13. In this discussion, we will summarize and discuss the most important results, and give some recommendations.

**Table 13.** An overview of the strengths and weaknesses of each strategy discussed^1^

		$r_{pc}$							
		dom^2^	epis	G × E	LD	Imprinting	Pedigree	Investment	Information
Purebred data	Additive model	-	-	-	+	-	no	+	+
	Dominance model	+	-	-	+	-	no	+	+
	Test in CB env.	-	-	+	+	-	no	0	+
Crossbred data	CB phenotypes	0	0	+	+	-	yes	-	+
	CB pheno + geno	0	0	+	-	-	no	- -	+
	Dominance model	+	0	+	-	-	no	- -	+
	BOA model	+	+	+	+	+	no	- -	-
	Correlated effects	0	0	+	-	-	no	--	++

^1^The left two columns indicate the different strategies, i.e., what type of data is used (first column) and what genomic prediction model is used (second column). All other columns indicate relative strengths and weaknesses of the strategies concerning several factors that affect accuracy of genomic prediction for crossbred performance. For example, a + for the dominance model in the column ‘dom’ indicates that the dominance model may be beneficial because it accounts for dominance.

^2^dom = dominance, epis = epistasis, G × E = genotype by environment interaction, LD = linkage disequilibrium, pedigree = whether or not pedigree is required, investment = amount of investment required, information = amount of information used to estimate breeding values + = strength, 0 = neutral, - = weakness.

### Using only purebred data

#### Accounting for dominance with PB data

Results from simulations and real data showed that using a dominance model instead of an additive model can result in a higher $\rho_{CB}$ and a higher short-term response to selection. It is likely that this benefit is only observed when dominance is an important cause for $r_{pc}$ values lower than one. Hence, this benefit increases with larger dominance effects, and with larger differences in allele frequencies between parental lines, because both lead to a lower $r_{pc}$.

Using simulations, Esfandyari et al. (2015b, 2018) showed that the dominance model leads to larger response in the short term, and smaller response in the long term, compared with the additive model. To explain this phenomenon, we focus on short vs. long-term response to selection with the additive model vs. the dominance model. Consider a single locus that has an additive (a) and dominance (d) genetic effect, and there is no mutation. For illustration, we assume that the allele frequencies in the two parental lines are quite different, namely $p_{1}=0.9$ in line 1 and $p_{2}=0.1$ in line 2, where p is the frequency of the positive allele (Note that if the allele frequencies are similar in the two parental lines, the additive and dominance model lead to similar average effects (see equations (3) and (4)), and it is, therefore, unlikely that this locus contributes to differences in selection response between the models.). We will discuss the short- and long-term responses to selection from the additive and dominance model for three scenarios where the locus shows overdominance (d>a), complete dominance (d=a), or incomplete dominance (d<a). With overdominance (d>a), the genotypic value of the crossbreds is maximized when the locus is fixed for opposite alleles in the two lines (Supplementary Figure S1, left plot). This can only be realized with the dominance model, because only the dominance model allows selection in the opposite direction of the average effect in the focal line (Equation 4). Hence, with overdominance, the dominance model results in greater short- and long-term responses than the additive model. With complete dominance (d=a), the genotypic value of the crossbreds is maximized when the positive allele is fixed in at least one of the parental lines (Supplementary Figure S1, middle plot). Fixation of the favorable allele can be realized the fastest in line 1, in which selection on this allele is much stronger with the dominance model (Equation 4) than with the additive model (Equation 3), resulting in a larger short-term response to selection with the dominance model. With incomplete dominance (d<a), the genotypic value of the crossbreds is maximized when the positive allele is fixed in both lines (Supplementary Figure S1, right plot). The probability that the favorable allele is lost in line 2 is greater with the dominance model compared with the additive model, because selection on this allele is weaker with the dominance model (Equation 4) than with the additive model (Equation 3). As a result, for loci with incomplete dominance, the probability that the positive allele becomes fixed in both lines is smaller with the dominance model than with the additive model, resulting in a smaller long-term response to selection.

In summary, the additive model may be beneficial for long-term response to selection for loci that exhibit incomplete dominance, whereas the dominance model may be beneficial for both short- and long-term responses to selection for loci that show complete or overdominance. It is important to note that in the aforementioned example and in the simulations of Esfandyari et al. (2018), there is no epistasis and no mutation. In reality, with epistasis, average effects are a function of allele frequencies at multiple loci (Cockerham, 1954; Kempthorne, 1954), making it difficult to predict how average effects change due to selection, and how those changes affect the difference in selection response between the additive and dominance model. Furthermore, the observed benefit of the additive model for long-term response to selection was due to the loss of favourable alleles in one of the parental lines (Esfandyari et al., 2018). In reality, new favorable alleles may appear in the population by mutation, possibly reducing the disadvantage of the dominance model for long-term response to selection. It is, therefore, not yet clear whether the benefit of the additive over the dominance model for long-term response to selection will be observed in real data.

#### Testing PB animals in a CB environment

With a fixed total testing capacity, testing a proportion of PB animals in a commercial environment reduces selection intensity, because those PB animals will not be allowed to return to the nucleus environment for biosecurity reasons. This strategy, therefore, results in a trade-off between the advantage of accounting for G × E, and the disadvantage of reduced selection intensity.

In their simulations of a broiler breeding program, Chu et al. (2018) showed that genomic prediction accuracy and response to selection were optimal when testing 30% of the PB selection candidates in the commercial environment (C). It is likely that this result depends on the total number of tested selection candidates, because accuracy and selection intensity show a diminishing return with the number of animals tested in C. As a result, with a very large number of candidates, the optimal fraction tested in C may be smaller than 30%, because additionally testing animals in C has a small impact on accuracy and selection intensity.

Testing PB animals in a CB environment may have an advantage over using a CB reference population because of differences in genetic relatedness with the selection candidates. For example, with a 4-way crossbreeding program without any multiplication steps, the available CB reference animals are separated by five generations from the selection candidates, whereas PB reference animals tested in a CB environment are separated by only two generations (Wientjes et al., 2020). As a result, the genetic relatedness between the reference animals and selection candidates is stronger when PB are tested in a CB environment, compared with when CB information is used, resulting in a higher expected accuracy.

In conclusion, genomic prediction accuracy and response to selection in CB performance can be improved without collecting data on crossbreds, but by testing PB animals in a commercial environment, especially when G × E is the most important reason for $r_{pc}$ values lower than about 0.8.

### Using crossbred data

Replacing PB by CB phenotypes in the reference population can improve accuracy and response to selection in CB performance because it accounts for $r_{pc}$ being smaller than one. A disadvantage of using CB phenotypes (without genotyping those crossbreds) is that it requires that the pedigree of these CB animals is available, while in the current practice, the pedigree of CB animals is usually not recorded. In addition, the CB animals need to be closely related to the selection candidates. In contrast to phenotypes of closely related PB animals, phenotypes of closely related CB animals are not always available at the time of selection. For example, in a pig breeding program where dams are selected before they have a recorded phenotype, the closest related PB animals with phenotypes are their parents (i.e., with an additive genetic relationship of 1/2 to the selection candidates), while the most related CB animals with phenotypes are the half-sibs of these parents (i.e., with an additive genetic relationship of 1/8 to the selection candidates). Finally, this strategy requires systematic phenotyping in crossbreds which may be costly. As a result, using only CB phenotypes for the estimation of BV for CB performance may currently not be practically feasible for most CB breeding programs.

The need for a recorded pedigree of phenotyped crossbreds can be alleviated by genotyping these animals. In addition, this strategy may improve genomic prediction accuracy, because predictions are based on actual genotypes of crossbreds, rather than on their genotype probabilities. Although the crossbreds with phenotypes and genotypes do not have to be closely related to the selection candidates, the accuracy is higher when this relationship is stronger (Wientjes et al., 2020). Hence, the benefit of collecting CB information partly depends on the genetic relatedness of the available CB reference animals to the selection candidates. The results from simulation and empirical data suggested that genotyping CB animals that already have phenotypes can improve genomic prediction accuracy and response to selection in CB performance when $r_{pc}$ is smaller than about 0.9. This benefit became larger with lower values of $r_{pc}$, and with a smaller number of genotyped and phenotyped purebreds. In conclusion, although phenotyping crossbreds may improve accuracy and selection response in some cases, it is advisable to genotype these crossbreds as well to optimally benefit from their recorded phenotypes.

Although a CB reference population accounts for $r_{pc}$ values lower than one, it has been argued that, e.g., each two-way CB record is only half as informative for selection in a PB parental line, compared with a PB record (Van Grevenhof and Van Der Werf, 2015). This may indeed be the case if the parental lines are completely unrelated, and the LD phase between these lines is uncorrelated. However, if the parental lines are somewhat related and the correlation of LD phase is larger than zero, the relative value of a CB record may be larger. Hence, the benefit of a CB reference population over a PB reference population probably depends on the relatedness between parental lines. Furthermore, for a trait with an $r_{pc}$ equal to 1, a CB reference population may even be at a disadvantage. It was shown that this disadvantage increases when the difference in LD between parental lines increased as the lines became more distantly related (Ibañez-Escriche et al., 2009). Finally, a single CB record can contribute to increased selection response in all parental lines, whereas a single PB record generally contributes to response in one parental line.

In summary, results from simulation and real data suggest that replacing a PB with a CB reference population is beneficial in terms of accuracy and response to selection, for traits with an $r_{pc}$ lower than about 0.8, when the PB and CB reference populations are of similar size, and when the CB animals in the reference population are at least moderately related to the selection candidates.

#### Accounting for dominance with CB data

Although results suggested that using a dominance model in a PB reference population is beneficial for accuracy and response to selection, this benefit was not observed with a CB reference population. An explanation for this result may be seen from the differences in average effects between the additive and dominance model. With a PB reference population, the difference in average effects between the additive model and dominance model is proportional to twice the allele frequency difference between lines (i.e., $2(p_{2}-p_{1})$), whereas with a CB reference population this difference is proportional to $\left(p_{2}-p_{1}\right)$. As a result, with a CB reference population, there is less gain in accuracy by using the dominance model instead of the additive model, compared with a PB reference population.

The number of studies that investigated the use of a dominance model with CB information is limited. Furthermore, most of these studies obtained accuracies from model reliabilities or by validating with total genetic values of CB animals. Such accuracies may not be comparable to accuracies of GEBV for CB performance in PB selection candidates. Nevertheless, the results suggest that the dominance model was at least as accurate as the additive model with both PB and CB data, even though it requires the estimation of twice as many effects. This is in line with studies that compared the performance of the dominance model with the additive model in genomic prediction within PB populations (e.g., Ertl et al., 2014; Heidaritabar et al., 2016; Duenk et al., 2017; Moghaddar and van der Werf, 2017). Furthermore, the dominance model may be interesting because average effects estimated in a CB reference population can be updated every generation by using current allele frequencies in the parental lines. It may, therefore, be interesting to study the robustness of the dominance model in more detail.

#### Considering the breed-origin of alleles in crossbreds

When the reference population includes phenotypes and genotypes of CB animals, the model can be refined by considering the breed-origin of alleles. This approach (called the BOA model) allows for breed-specific marker effects due to 1) dominance (or some types of epistasis) in combination with differences in allele frequencies between parental lines, and 2) differences in LD phase between parental lines. However, as was shown by Ibañez-Escriche et al. (2009) and Zeng et al. (2013), a difference in LD phase between parental lines alone did not result in a benefit of the BOA model when marker density was high and breeds were somewhat related. In empirical data, the BOA model seemed to be beneficial when $r_{pc}$ was smaller than about 0.8, suggesting that non-additive effects in combination with differences in allele frequencies between parental lines makes an important contribution to the benefit of the BOA model.

When the BOA is considered, the marker alleles in crossbreds coming from one parental line do not contribute to the estimation of marker effects for the other parental line. However, alleles from one parental line can be valuable for estimation of effects in another parental line when the effects of marker alleles in crossbreds do not strongly depend on breed-origin. Such a situation can occur when the allele frequencies and LD phase between the parental lines are correlated. Considering the BOA in those cases may result in the removal of valuable information for the estimation of marker effects for CB performance, leading to a disadvantage of considering the BOA. The benefit of considering the BOA depends on the balance between the advantage of accounting for breed-specific effects, and the disadvantage of removing valuable information.

For many cases discussed in this review, ignoring the BOA was preferred over considering the BOA, suggesting that the disadvantage of reduced information to estimate markers effects is often greater than the advantage of allowing for breed-specific effects. This explanation for the competitive performance of the additive model in a CB reference population is in agreement with results of our study on body weight in broiler chicken (Duenk et al., 2019b). In that study, we observed that ignoring the BOA was beneficial for a trait with an $r_{pc}$ of 0.96. This result suggests that, although parental lines were believed to be distantly related, the LD between markers and QTL was similar in the parental lines. To further test this hypothesis, we estimated GEBV of the sires using a CB reference population where only the alleles from the dam line were considered. We expected that these GEBV would be uncorrelated with GEBV resulting from a reference population of PB animals from the sire line, because the alleles used to estimate marker effects originated from different parental lines. The results showed, however, that the correlation between these GEBVs was ~0.15, suggesting that the LD phase between parental lines was somewhat similar (see also Duenk, 2020). Hence, the advantage of considering the BOA to account for breed-specific effects was probably smaller than the disadvantage of removing valuable information to estimate marker effects.

In summary, it seems that the possible benefit of the BOA model primarily comes from differences in allele frequencies between parental lines in the presence of dominance (and possibly epistatic interactions that lead to breed-specific effects). On the one hand, this benefit may only be observed with a relatively large CB reference population, because the BOA model possibly utilizes less information than the additive model when parental lines are somewhat related. On the other hand, the benefit of the BOA model may disappear with a relatively large CB reference population, because the model parameterization (i.e., the prior) can be overwhelmed by the data.

#### Allowing for correlated effects between parental lines and the crossbreds

A disadvantage of considering the BOA in the crossbreds is the assumption of uncorrelated genetic effects across the parental lines. In reality, however, genetic effects may be correlated across parental lines, which are reflected by non-zero values of genetic correlations between populations. In such situations, considering the BOA may not be beneficial, and it may be advisable to allow for correlated effects across all populations (Christensen et al., 2015; Vitezica et al., 2016). Although comparisons between models that assume either correlated or uncorrelated genetic effects across parental lines are very limited, the correlated effects model may be beneficial when parental lines are not distantly related, and when effects of alleles in the crossbreds do not depend on breed-of-origin.

#### A note on imprinting

Imprinting is the phenomenon where the effect of a transmitted allele depends on whether it is inherited paternally or maternally, so that reciprocal heterozygotes have different genotypic values. In crossbreeding programs such as those of pigs and poultry, paternal alleles may always be inherited from the same breed, and maternal alleles from another breed. As a result, imprinting may lead to apparent breed-specific effects of alleles in CB animals. With any reference population, imprinting can be accounted for in genomic models when the parental origin of alleles is known (e.g., Esfandyari et al., 2015a; Nishio and Satoh, 2015; Hu et al., 2016). For CB reference populations, a genomic model that accounts for both dominance and imprinting was discussed by Stock et al. (2020). Theoretically, this model results in the same average effects for CB performance as the BOA model. Hence, both models account for breed-specific effects due to imprinting, differences in LD, and due to dominance in combination with differences in allele frequencies.

In reality, the BOA model may have an advantage over the dominance and imprinting model, because the BOA model it can account for epistasis as well. In addition, estimating imprinting in pig and poultry breeding can be challenging because data on reciprocal crosses are usually not available. At the same time, however, the BOA model may have a disadvantage because the amount of information for the estimation of marker effects may be smaller (see section on Considering the breed-origin of alleles). So far, there have been no studies that looked at the difference in performance between a model that accounts for the BOA, and a model that accounts for dominance and (possibly) imprinting.

### Relationship between accuracy and response to selection

This review focussed on differences in accuracy and response to selection between strategies that aim to improve CB performance with genomic selection. When breeding values and phenotypes follow a multivariate normal distribution, it follows from the breeder’s equation that a difference in accuracy between two strategies results in an identical relative difference in short-term response to selection. However, strategies may also differ in the way they exploit additive genetic variance of CB performance, resulting in a difference in response to selection in the long term. One example is the use of a dominance instead of an additive model in a PB reference population. Compared with the additive model, there is a smaller chance that rare favorable alleles are lost due to drift with the dominance model, preserving more genetic variation in subsequent generations (see “Accounting for dominance”). So, it is important to note that differences in long-term response to selection between selection strategies are not only a function of differences in accuracy, but of differences in the utilization of additive genetic variance of CB performance as well.

### Model complexity

This review focussed on differences in accuracy and response to selection in CB performance between models. Other important aspects for breeders that use genomic prediction are computation time and model convergence. Although these aspects were not compared in any of the studies presented in this review, it is expected that computation time increases as the model becomes more complex (i.e., as the number of parameters that need to be estimated increases). For example, it is expected that the dominance model requires about twice as much computation time as the additive model, because the number of parameters is twice as large. Furthermore, the number of iterations needed to reach convergency is likely to increase with increasing model complexity. Finally, assigning the BOA in crossbreds is not a trivial task, and is computationally demanding (Sevillano et al., 2016; Vandenplas et al., 2016). The decision to use a more complex model may, therefore, depend on whether the expected benefit in accuracy and response to selection justifies increased computational requirements.

### Recommendations

#### Comparing strategies

The choice of strategy to compute GEBV for CB performance of PB animals is of great practical relevance for pig and poultry breeders. Researchers that aim to compare such strategies, therefore, need to carefully scrutinize the differences between their study and the practical situation. For example, in practice it is likely that the PB selection candidates are more closely related to a PB reference population than to a CB reference population (Wientjes et al., 2020). It is advisable to reflect this difference in the data used or simulated when studying the benefit of a CB over a PB reference population. When some aspects of the study do not match with the practical situation, the study should at least report these disparities and discuss their impact on the results. In addition, we recommend that studies report the $r_{pc}$ and heritability of the studied trait, describe the relationships between the reference population(s) and selection candidates, and describe or estimate the genetic distance between the parental lines.

#### Choice of strategy

This review showed that the differences in accuracy and response to selection between strategies depend on several factors. Many of these factors are properties of the trait of interest (such as heritability and $r_{pc}$), or of the structure of the breeding program (such as the distance between reference animals and selection candidates). Because these properties can vary substantially, the optimal strategy varies across scenarios. Breeders would, therefore, benefit from a tool that allows them to predict the response to selection from different strategies, for their specific scenario. Such a tool requires that the accuracy of GEBV for CB performance can be predicted. For instance, Wientjes et al. (2020) showed that it is possible to predict the accuracy of strategies that use an additive model while considering the BOA, based on $r_{pc}$, the size of the reference population, the heritability of the trait, and the effective number of independent chromosome segments between the reference population and selection candidates. We argue that future research should focus on the development of a tool that predicts accuracy and response to selection from scenario specific parameters, instead of focussing on empirical comparisons between strategies that use different prediction models or reference populations.

The differences in response to selection between strategies largely depend on differences in accuracies of GEBV for CB performance. In turn, these differences in accuracies largely depend on the value of $r_{pc}$, especially when the reference populations are large. We, therefore, recommend obtaining accurate estimates of $r_{pc}$ of all breeding goal traits. Furthermore, knowledge about the importance of components of $r_{pc}$ (i.e., G × G and G × E) may help breeders to decide which model they should use, and whether they should systematically collect data on animals in a CB environment or not. For example, when G × E is unimportant, testing PB animals in a CB environment is not recommended. Although hardly any studies have tried to quantify the contribution of these different components to $r_{pc}$, a comparison of estimated $r_{pc}$ across studies suggested that the contribution of G × E is likely to be smaller than the contribution of G × G (Wientjes and Calus, 2017).

When $r_{pc}$ is higher than about 0.9, collecting CB information to account for $r_{pc}<1$ may not outweigh the disadvantage of the increased distance of the CB reference population to the selection candidates, compared with a PB reference population. With a PB reference population, the use of a dominance model may be advantageous, because it yields accuracies that are equal to or higher than accuracies yielded by additive models.

When $r_{pc}$ is lower than about 0.8, and is caused mainly by G × E, it may be beneficial to create a reference population of PB animals that are tested in a CB environment. A benefit of this strategy is that the genetic relatedness between selection candidates and the reference population is usually stronger with a PB than with a CB reference population. It should be kept in mind, however, that a single PB record only provides information for estimation of GEBV for one parental line, whereas a single CB record provides information for all parental lines. Furthermore, it may be interesting to investigate the benefit of the dominance model in a reference population of PB animals that were tested in a commercial environment. Such a strategy may account for both dominance and G × E, without the need for collecting data on CB animals.

When $r_{pc}$ is lower than about 0.8, and is caused by both G × G and G × E, collecting information of CB animals may be beneficial. Collecting only CB phenotypes may slightly improve accuracy and response to selection, but requires that the pedigree is known. It is, therefore, advisable to genotype these CB animals as well. Although collecting CB phenotypes and genotypes may be costly, a single CB record can contribute to increased accuracy and selection response in all parental lines, whereas a single PB record generally contributes to response in one parental line. Investing in a CB reference population may, therefore, be advantageous, especially for traits with low $r_{pc}$. It should be kept in mind, however, that a CB reference population may be less related to the selection candidates than a PB reference population, thereby reducing the benefit of using CB instead of PB records. Finally, considering the BOA in a CB reference population may be beneficial, because such a model allows for breed-specific effects of alleles in the crossbreds (Duenk, 2020). However, the BOA model may not always lead to higher accuracies of GEBV, because its advantages may be overshadowed by its disadvantage of using less information to estimate marker effects, especially in small reference populations. In those cases, it may be beneficial to use a genomic prediction model with genetic effects that are correlated across each parental line and the crossbreds.

## Supplementary Material

skab205_suppl_Supplementary_Materials

## Abbreviations

A-DI: additive + dominance + imprinting model
A-D: additive + dominance model
A: additive model
A-C: additive model with correlated genetic effects across parental lines
A-NOOP: additive model without own performance records
ADFI: average daily feed intake
ADG: average daily gain
BF: backfat thickness
BLUP: best linear unbiased prediction
A-BOA: BOA model
BW35: body weight at 35 days
BW7: body weight at 7 days
BV: breeding values
BOA: breed-origin of alleles
C: commercial environment
CB: crossbred
DEBV: deregressed EBV
DL: Dutch Landrace
EBV: estimated breeding values
gSD: genetic standard deviations
GBLUP: genomic best linear unbiased prediction
GEBV: genomic estimated breeding values
GxE: genotype by environment interaction
GxG: genotype by genotype interaction
GLE: gestation length
LL: Landrace
LW: Large White
LPL: length of productive life
LD: linkage disequilibrium
LS: litter size
LDP: loin depth
MAS: marker assisted selection
MS: marker selection
NSB: number stillborn
PBA: piglets born alive
PB: purebred
PEV: prediction error variance
$r_{pc}$: purebred-crossbred genetic correlation
QTL: quantitative trait loci
SS-A: singel-step additive model
TNB: total number born
YY: Yorkshire
