# Supplementary material for: Review: optimizing genomic selection for crossbred performance by model improvement and data collection
Source: J Anim Sci. 2021 Jul 5;99(8):skab205. doi: 10.1093/jas/skab205 (PMC8499581; doi:10.1093/jas/skab205)
Supplement: skab205_suppl_Supplementary_Materials [file skab205_suppl_supplementary_materials.docx]

**Supplementary Figures**


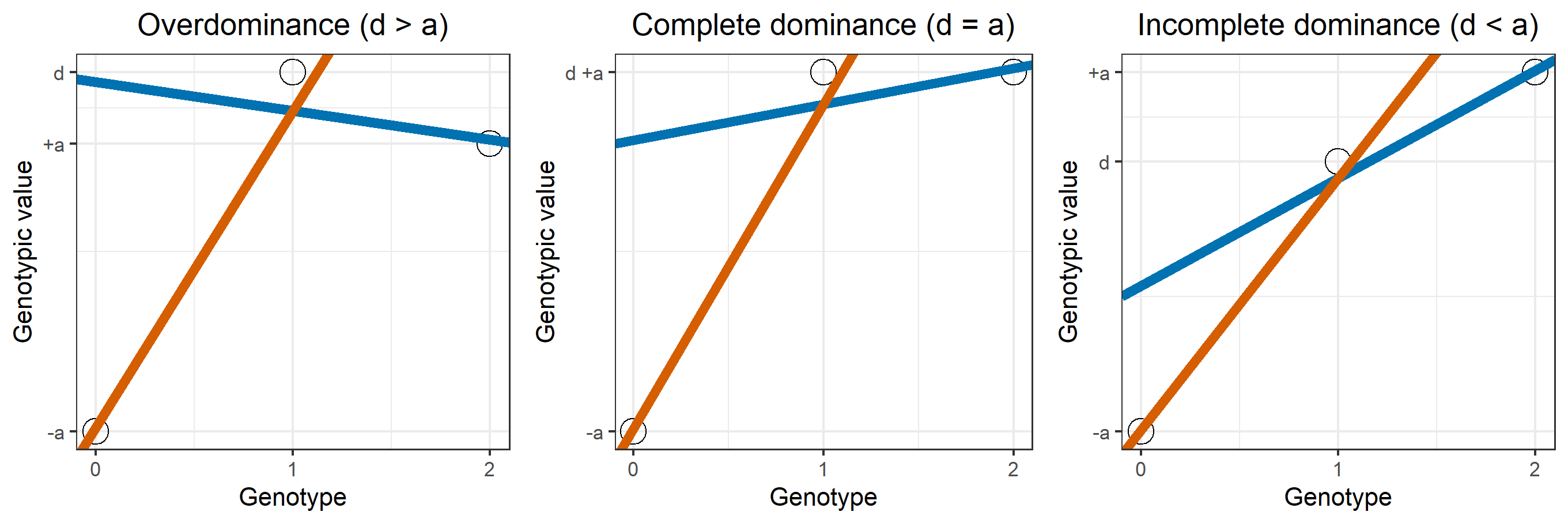


*Figure S 1 Average effects shown as regressions of genotypic value on allele counts (i.e., genotype). The three panels indicate if there is overdominance, complete dominance, or incomplete dominance. Open circles show the genotypic values, and lines are regression lines with average effects as the slope. The blue lines show average effects from the additive model in line 1 or the dominance model in line 2, while the orange lines show the average effects from dominance model in line 1 or the additive model in line 2. The frequency of the counted allele was 0.9 in line 1, and 0.1 in line 2.*

**Supplementary Tables**

*Table S 1 Change in accuracy (*$\Delta_{\rho}$*) and response to selection in CB performance (*$\Delta_{R}$*) when a fraction of the PB animals were tested in a commercial environment in each generation, compared to when all PB animals were tested in the nucleus environment.*

| study | data | line | Model | h^2^ | r_pc_ | N_PB_ | % tested in C | a_PB_ | a_CB_ | $\Delta_{\rho}$ | $\Delta_{R}$ |
| --- | --- | --- | --- | --- | --- | --- | --- | --- | --- | --- | --- |
| Chu2018 | broilers | L-H | PB sire line | SS-A | 0.5 | 1280 | 30 | 0-1 | 0-0.5 |  | 0.10 |
| Chu2018 | broilers | L-L | PB sire line | SS-A | 0.5 | 1280 | 30 | 0-1 | 0-0.5 |  | 0.08 |
| Chu2018 | broilers | L-M | PB sire line | SS-A | 0.5 | 1280 | 30 | 0-1 | 0-0.5 | 0.41 | 0.09 |
| Chu2018 | broilers | M-H | PB sire line | SS-A | 0.7 | 1280 | 30 | 0-1 | 0-0.5 |  | 0.04 |
| Chu2018 | broilers | M-L | PB sire line | SS-A | 0.7 | 1280 | 30 | 0-1 | 0-0.5 |  | 0.04 |
| Chu2018 | broilers | M-M | PB sire line | SS-A | 0.7 | 1280 | 30 | 0-1 | 0-0.5 | 0.26 | 0.05 |
| Chu2018 | broilers | H-H | PB sire line | SS-A | 0.9 | 1280 | 30 | 0-1 | 0-0.5 |  | 0.01 |
| Chu2018 | broilers | H-L | PB sire line | SS-A | 0.9 | 1280 | 30 | 0-1 | 0-0.5 |  | 0.00 |
| Chu2018 | broilers | H-M | PB sire line | SS-A | 0.9 | 1280 | 30 | 0-1 | 0-0.5 | 0.07 | 0.01 |
| Chu2020 | trout | T1 | PB line B | SS-A | 0.5 | 1000 | 20 | 0-0.125 | 0-0.125 | 0.29 | 0.09 |
| Chu2020 | trout | T1 | PB line B | SS-A | 0.5 | 1000 | 40 | 0-0.125 | 0-0.125 | 0.38 | 0.08 |
| Chu2020 | trout | T1 | PB line B | SS-A | 0.5 | 1000 | 60 | 0-0.125 | 0-0.125 | 0.44 | 0.06 |
| Chu2020 | trout | T2 | PB line B | SS-A | 0.8 | 1000 | 20 | 0-0.125 | 0-0.125 | 0.14 | 0.02 |
| Chu2020 | trout | T2 | PB line B | SS-A | 0.8 | 1000 | 40 | 0-0.125 | 0-0.125 | 0.20 | 0.01 |
| Chu2020 | trout | T2 | PB line B | SS-A | 0.8 | 1000 | 60 | 0-0.125 | 0-0.125 | 0.26 | 0.00 |

*^1^Accuracies were only reported for scenarios with a heritability for CB performance of 0.25.
N_PB_ denotes the number of PB selection candidates that were phenotyped and genotyped in each generation.*

*Table S 2 Change in accuracy (*$\Delta_{\rho}$*) and response to selection in CB performance (*$\Delta_{R}$*) when PB phenotypes are replaced with CB phenotypes, when only PB genotypes are available.*

| study | Data | trait | line | model | r_pc_ | N_PB_ | p_PB;_ p_CB_ | a_PB_ | a_CB_ | $\Delta_{\rho}$ | $\Delta_{R}$ |
| --- | --- | --- | --- | --- | --- | --- | --- | --- | --- | --- | --- |
| Esfandyari2015a | stoch | T1 | PB line | A-D | 0.78 | 2000 | 0;1 | 0-0.5 | 0-0.125 |  | 0.03 |
| Esfandyari2015a | stoch | T1 | PB line | A-D-BOA | 0.78 | 2000 | 0;1 | 0-0.5 | 0-0.125 |  | 0.06 |
| See2020 | stoch | T1 | PB sire line | SS-A | 0.3 | 2100 | 0;0.76 | 0-1 | 0-0.25 | 0.09 | 0.29 |
| See2020 | stoch | T2 | PB sire line | SS-A | 0.7 | 2100 | 0;0.76 | 0-1 | 0-0.25 | 0.01 | 0.11 |
| See2020 | stoch | T3 | PB sire line | SS-A | 0.9 | 2100 | 0;0.76 | 0-1 | 0-0.25 | -0.13 | -0.06 |
| Tusell2020 | pig | ADG | PB sire line | A |  | 5137 | 0;0.54 | 0-0.5 | 0-0.25 | 0.09 |  |
| Tusell2020 | pig | RFI | PB sire line | A |  | 5137 | 0;0.54 | 0-0.5 | 0-0.25 | -0.17 |  |
| Tusell2020 | pig | ADG | PB sire line | SVM |  | 5137 | 0;0.54 | 0-0.5 | 0-0.25 | 0.17 |  |
| Tusell2020 | pig | RFI | PB sire line | SVM |  | 5137 | 0;0.54 | 0-0.5 | 0-0.25 | 0.04 |  |
| Tusell2020 | pig | ADG | PB sire line_young | A |  | 3209 | 0;0.95 | 0-0.5 | 0-0.25 | -0.21 |  |
| Tusell2020 | pig | RFI | PB sire line_young | A |  | 3209 | 0;0.95 | 0-0.5 | 0-0.25 | -0.11 |  |
| Tusell2020 | pig | ADG | PB sire line_young | SVM |  | 3209 | 0;0.95 | 0-0.5 | 0-0.25 | 0.07 |  |
| Tusell2020 | pig | RFI | PB sire line_young | SVM |  | 3209 | 0;0.95 | 0-0.5 | 0-0.25 | -0.16 |  |

*Table S 3 Change in accuracy (*$\Delta_{\rho}$*) and response to selection in CB performance (*$\Delta_{R}$*) when CB genotypes are added, for the scenario where only CB phenotypes are used.*

| study | sp | trait | line | model | r_pc_ | N_CB_ | p_geno_ | a_CB_ | $\Delta_{\rho}$ | $\Delta_{R}$ |
| --- | --- | --- | --- | --- | --- | --- | --- | --- | --- | --- |
| Esfandyari2015a |  | T1 | PB line | A-D | 0.78 | 2000 | 1.00 | 0-0.125 |  | 0.04 |
| Esfandyari2015a |  | T1 | PB line | A-D-BOA | 0.78 | 2000 | 1.00 | 0-0.125 |  | 0.04 |
| See2020 | pigs | T1 | PB sire line | SS-A | 0.3 | 1600 | 1.00 | 0-0.25 | 0.23 | 0.06 |
| See2020 | pigs | T2 | PB sire line | SS-A | 0.7 | 1600 | 1.00 | 0-0.25 | 0.17 | 0.14 |
| See2020 | pigs | T3 | PB sire line | SS-A | 0.9 | 1600 | 1.00 | 0-0.25 | 0.16 | 0.10 |
| Sewell2018 | pig | ADG | PB sire line | SS-A |  | 667 | 0.50 | unclear | 0.04 |  |
| Sewell2018 | pig | ADG | PB sire line | SS-A |  | 667 | 1.00 | unclear | 0.08 |  |
| Sewell2018 | pig | ADG | PB sire line | SS-A |  | 667 | 1.50 | unclear | 0.16 |  |
| Sewell2018 | pig | ADG | PB sire line | SS-A |  | 667 | 1.88 | unclear | 0.31 |  |
| Sewell2018 | pig | BF | PB sire line | SS-A |  | 667 | 0.50 | unclear | 0.06 |  |
| Sewell2018 | pig | BF | PB sire line | SS-A |  | 667 | 1.00 | unclear | 0.19 |  |
| Sewell2018 | pig | BF | PB sire line | SS-A |  | 667 | 1.50 | unclear | 0.28 |  |
| Sewell2018 | pig | BF | PB sire line | SS-A |  | 667 | 1.88 | unclear | 0.36 |  |
| Sewell2018 | pig | LDP | PB sire line | SS-A |  | 667 | 0.50 | unclear | 0.19 |  |
| Sewell2018 | pig | LDP | PB sire line | SS-A |  | 667 | 1.00 | unclear | 0.30 |  |
| Sewell2018 | pig | LDP | PB sire line | SS-A |  | 667 | 1.50 | unclear | 0.42 |  |
| Sewell2018 | pig | LDP | PB sire line | SS-A |  | 667 | 1.88 | unclear | 0.57 |  |
| Tusell2020 | pigs | ADG | PB sire line_young | A |  | 3059 | 1.31 | 0-0.25 | 0.05 |  |
| Tusell2020 | pigs | RFI | PB sire line_young | A |  | 3059 | 1.31 | 0-0.25 | 0.28 |  |
| Tusell2020 | pigs | ADG | PB sire line_young | SVM |  | 3059 | 1.31 | 0-0.25 | 0.16 |  |
| Tusell2020 | pigs | RFI | PB sire line_young | SVM |  | 3059 | 1.31 | 0-0.25 | 0.11 |  |

*Table S 4 Change in accuracy (*$\Delta_{\rho}$*) when CB genotypes are added, for the scenario where both PB and CB phenotypes are used.*

| Study | trait | line | model | r_pc_ | N_PB_ | p_PB_;p_CB_ | a_PB_ | a_CB_ | $\Delta_{\rho}$ | $\Delta_{R}$ |
| --- | --- | --- | --- | --- | --- | --- | --- | --- | --- | --- |
| Grevenhof2015 | T1 | PB line | A | 0.7 | 2000 | 0;3 |  |  | 0.05 |  |
| Grevenhof2015 | T1 | PB line | A | 0.7 | 2000 | 1;1 |  |  | 0.02 |  |
| Grevenhof2015 | T1 | PB line | A | 0.7 | 2000 | 1;2 |  |  | 0.04 |  |
| Grevenhof2015 | T1 | PB line | A | 0.7 | 6000 | 0;1 |  |  | 0.03 |  |
| Grevenhof2015 | T1 | PB line | A | 0.7 | 6000 | 0.5;0.5 |  |  | 0.01 |  |
| Grevenhof2015 | T3 | PB line | A | 0.5 | 6000 | 0;1 |  |  | 0.06 |  |
| Grevenhof2015 | T3 | PB line | A | 0.5 | 6000 | 0.5;0.5 |  |  | 0.03 |  |
| Grevenhof2015 | T2 | PB line | A | 0.9 | 6000 | 0;1 |  |  | *-0.01* |  |
| Grevenhof2015 | T2 | PB line | A | 0.9 | 6000 | 0.5;0.5 |  |  | *-0.01* |  |
| Grevenhof2015 | LPL | PB line | A-NOOP | 0.5 | 6000 | 0.5;0.5 |  |  | 0.02 |  |
| Grevenhof2015 | LPL | PB line | A-NOOP | 0.7 | 6000 | 0.5;0.5 |  |  | 0.00 |  |
| Grevenhof2015 | LPL | PB line | A-NOOP | 0.9 | 6000 | 0.5;0.5 |  |  | *-0.02* |  |
| Grevenhof2015 | PBA | PB dam line | A-NOOP | 0.5 | 4000 | 0.25;0.75 |  |  | 0.03 |  |
| Grevenhof2015 | PBA | PB dam line | A-NOOP | 0.7 | 4000 | 0.25;0.75 |  |  | 0.00 |  |
| Grevenhof2015 | PBA | PB dam line | A-NOOP | 0.9 | 4000 | 0.25;0.75 |  |  | *-0.01* |  |
| Grevenhof2015 | T1 | PB line | A-NOOP | 0.7 | 2000 | 1;1 |  |  | 0.08 |  |
| Grevenhof2015 | T1 | PB line | A-NOOP | 0.7 | 2000 | 1;2 |  |  | 0.14 |  |
| Xiang2017 | TNB | LL sires | A-C | 0.79 | 7800 | 1;0.67 | 0-1 | 0.5 | 0.09 |  |
| Xiang2017 | TNB | YY sires | A-C | 0.68 | 7800 | 1;0.67 | 0-1 | 0.5 | 0.03 |  |
| Sewell2018 | ADG | PB sire line | A |  | 667 | 1;0.5 |  |  | 0.08 |  |
| Sewell2018 | ADG | PB sire line | A |  | 667 | 1;1 |  |  | 0.04 |  |
| Sewell2018 | ADG | PB sire line | A |  | 667 | 1;1.5 |  |  | 0.08 |  |
| Sewell2018 | ADG | PB sire line | A |  | 667 | 1;1.88 |  |  | 0.14 |  |
| Sewell2018 | BF | PB sire line | A |  | 667 | 1;0.5 |  |  | *-0.01* |  |
| Sewell2018 | BF | PB sire line | A |  | 667 | 1;1 |  |  | 0.03 |  |
| Sewell2018 | BF | PB sire line | A |  | 667 | 1;1.5 |  |  | 0.05 |  |
| Sewell2018 | BF | PB sire line | A |  | 667 | 1;1.88 |  |  | 0.06 |  |
| Sewell2018 | LDP | PB sire line | A |  | 667 | 1;0.5 |  |  | 0.09 |  |
| Sewell2018 | LDP | PB sire line | A |  | 667 | 1;1 |  |  | 0.09 |  |
| Sewell2018 | LDP | PB sire line | A |  | 667 | 1;1.5 |  |  | 0.12 |  |
| Sewell2018 | LDP | PB sire line | A |  | 667 | 1;1.88 |  |  | 0.19 |  |
| See2020 | T1 | PB sire line | SS-A | 0.3 | 2100 | 1;0.38 | 0-1 | 0-0.25 | 0.12 | 0.12 |
| See2020 | T1 | PB sire line | SS-A | 0.3 | 2100 | 1;0.76 | 0-1 | 0-0.25 | 0.20 | 0.17 |
| See2020 | T2 | PB sire line | SS-A | 0.7 | 2100 | 1;0.38 | 0-1 | 0-0.25 | 0.08 | 0.05 |
| See2020 | T2 | PB sire line | SS-A | 0.7 | 2100 | 1;0.76 | 0-1 | 0-0.25 | 0.15 | 0.10 |
| See2020 | T3 | PB sire line | SS-A | 0.9 | 2100 | 1;0.38 | 0-1 | 0-0.25 | 0.01 | -0.09 |
| See2020 | T3 | PB sire line | SS-A | 0.9 | 2100 | 1;0.76 | 0-1 | 0-0.25 | 0.05 | -0.07 |
| Sevillano2018 | ADFI | PB sire line | SS-A | 0.75-0.88 | 6594 | 1;0.45 | 0.5 | 0-0.25 | 0.08 |  |
| Sevillano2018 | ADG | PB sire line | SS-A | 0.75 | 6594 | 1;0.45 | 0.5 | 0-0.25 | 0.09 |  |
| Sevillano2018 | BF | PB sire line | SS-A | 0.8 | 6594 | 1;0.45 | 0.5 | 0-0.25 | 0.04 |  |
| Sevillano2018 | LDP | PB sire line | SS-A | 0.75-0.88 | 6594 | 1;0.45 | 0.5 | 0-0.25 | 0.00 |  |

*All studies presented in this table were based on simulations, except for Sevillano2018, which was on pigs.*

*Table S 5 Change in accuracy (*$\Delta_{\rho}$*) and response to selection in CB performance (*$\Delta_{R}$*) when PB phenotypes and genotypes are replaced by CB phenotypes and genotypes (i.e. replacing a PB reference population with a CB reference population).*

| study | data | trait | line | model | r_pc_ | N_PB_ | N_CB_ | a_PB_ | a_CB_ | $\Delta_{\rho}$ | $\Delta_{R}$ |
| --- | --- | --- | --- | --- | --- | --- | --- | --- | --- | --- | --- |
| Dekkers2007 | det | T1 |  | MAS | 0.7 |  |  |  |  | 0 | 0.02 |
| Dekkers2007 | det | T2 |  | MAS | 0.7 |  |  |  |  | 0 | 0.12 |
| Dekkers2007 | det | T3 |  | MAS | 0.7 |  |  |  |  | 0 | 0.30 |
| Dekkers2007 | det | T1 |  | MS | 0.7 |  |  |  |  | 0 | 0.07 |
| Dekkers2007 | det | T2 |  | MS | 0.7 |  |  |  |  | 0 | 0.17 |
| Dekkers2007 | det | T3 |  | MS | 0.7 |  |  |  |  | 0 | 0.31 |
| Ibanez-Escriche2009 | stoch | T1 | 3w dam | A | 1 | 4000 | 4000 | 0 | 0 | *-0.09* |  |
| Ibanez-Escriche2009 | stoch | T1 | 3w sire | A | 1 | 4000 | 4000 | 0 | 0 | *-0.11* |  |
| Ibanez-Escriche2009 | stoch | T1 | 4w PB | A | 1 | 4000 | 4000 | 0 | 0 | *-0.09* |  |
| Ibanez-Escriche2009 | stoch | T1 | 4w PB UR^1^ | A | 1 | 4000 | 4000 | 0 | 0 | *-0.22* |  |
| Kinghorn2010 | stoch | T1 |  | A |  | 400 | 400 |  |  |  | 0.07 |
| Esfandyari2015a | stoch | T1 | PB line | A-D | 0.78 | 2000 | 2000 | 0-0.5 | 0-0.125 | 0.12 | 0.08 |
| Hidalgo2016 | pig | GLE | DL line | A | 0.94 | 550 | 550 | 0.04 | 0.03 | *-0.14* |  |
| Hidalgo2016 | pig | GLE | LW line | A | 0.94 | 550 | 550 | 0.04 | 0.03 | *-0.03* |  |
| Hidalgo2016 | pig | TNB | DL line | A | 0.9 | 914 | 914 | 0.04 | 0.03 | *-0.15* |  |
| Hidalgo2016 | pig | TNB | LW line | A | 0.9 | 914 | 914 | 0.04 | 0.03 | *-0.18* |  |
| Esfandyari2018 | stoch | T1-G40 | PB sire line | A | 0.82 | 2000 | 2000 | 0-0.5 | 0-0.25 |  | 0.02 |
| Esfandyari2018 | stoch | T1-G5 | PB sire line | A | 0.82 | 2000 | 2000 | 0-0.5 | 0-0.25 | 0.07 | 0.09 |
| Duenk2019 | broilers | BW35 | PB sire line | A | 0.96 | 4471 | 4445 | 0-0.125 | 0-0.125 | *-0.10* |  |
| Duenk2019 | broilers | BW7 | PB sire line | A | 0.8 | 4687 | 4655 | 0-0.125 | 0-0.125 | 0.00 |  |
| See2020 | stoch | T1 | PB sire line | SS-A | 0.3 | 2100 | 1600 | 0-1 | 0-0.25 | 0.32 | 0.34 |
| See2020 | stoch | T2 | PB sire line | SS-A | 0.7 | 2100 | 1600 | 0-1 | 0-0.25 | 0.18 | 0.25 |
| See2020 | stoch | T3 | PB sire line | SS-A | 0.9 | 2100 | 1600 | 0-1 | 0-0.25 | 0.03 | 0.04 |
| Tusell2020 | pigs | ADG | PB sire line | A |  | 3209 | 3998 |  |  | 0.10 |  |
| Tusell2020 | pigs | RFI | PB sire line | A |  | 3209 | 3998 |  |  | 0.04 |  |
| Tusell2020 | pigs | ADG | PB sire line | SVM |  | 3209 | 3998 |  |  | 0.15 |  |
| Tusell2020 | pigs | RFI | PB sire line | SVM |  | 3209 | 3998 |  |  | 0.08 |  |
| Tusell2020 | pigs | ADG | PB sire line_young | A |  | 3209 | 3998 |  |  | -0.16 |  |
| Tusell2020 | pigs | RFI | PB sire line_young | A |  | 3209 | 3998 |  |  | 0.17 |  |
| Tusell2020 | pigs | ADG | PB sire line_young | SVM |  | 3209 | 3998 |  |  | 0.23 |  |
| Tusell2020 | pigs | RFI | PB sire line_young | SVM |  | 3209 | 3998 |  |  | -0.05 |  |

*^1^UR = unrelated parental lines*

*Table S 6 Change in accuracy (*$\Delta_{\rho}$*) and response to selection in CB performance (*$\Delta_{R}$*) when CB phenotypes and genotypes are added to a PB reference population.*

| study | data | trait | line | model | r_pc_ | N_PB_ | p_PB_;p_CB_ | a_PB_ | a_CB_ | $\Delta_{\rho}$ | $\Delta_{R}$ |
| --- | --- | --- | --- | --- | --- | --- | --- | --- | --- | --- | --- |
| Gonzalez-Dieguez2020 | stoch | T1 | PB line | A-D | 0.46 | 2032 | 1;1 | 0-0.5 | 0-0.125 |  | 0.17 |
| Gonzalez-Dieguez2020 | stoch | T2 | PB line | A-D | 0.3 | 2032 | 1;1 | 0-0.5 | 0-0.125 |  | 0.37 |
| Gonzalez-Dieguez2020 | stoch | T3 | PB line | A-D | 0.42 | 2032 | 1;1 | 0-0.5 | 0-0.125 |  | 0.30 |
| Gonzalez-Dieguez2020 | stoch | T4 | PB line | A-D | 0.68 | 2032 | 1;1 | 0-0.5 | 0-0.125 |  | 0.18 |
| See2020 | stoch | T1 | PB line | SS-A | 0.3 | 2100 | 0.5;0.38 | 0-1 | 0-0.25 | 0.21 | 0.32 |
| See2020 | stoch | T1 | PB line | SS-A | 0.3 | 2100 | 1;0.38 | 0-1 | 0-0.25 | 0.26 | 0.39 |
| See2020 | stoch | T1 | PB line | SS-A | 0.3 | 2100 | 1;0.76 | 0-1 | 0-0.25 | 0.34 | 0.44 |
| See2020 | stoch | T2 | PB line | SS-A | 0.7 | 2100 | 0.5;0.38 | 0-1 | 0-0.25 | 0.15 | 0.29 |
| See2020 | stoch | T2 | PB line | SS-A | 0.7 | 2100 | 1;0.38 | 0-1 | 0-0.25 | 0.15 | 0.25 |
| See2020 | stoch | T2 | PB line | SS-A | 0.7 | 2100 | 1;0.76 | 0-1 | 0-0.25 | 0.22 | 0.29 |
| See2020 | stoch | T3 | PB line | SS-A | 0.9 | 2100 | 0.5;0.38 | 0-1 | 0-0.25 | 0.02 | 0.14 |
| See2020 | stoch | T3 | PB line | SS-A | 0.9 | 2100 | 1;0.38 | 0-1 | 0-0.25 | 0.05 | 0.25 |
| See2020 | stoch | T3 | PB line | SS-A | 0.9 | 2100 | 1;0.76 | 0-1 | 0-0.25 | 0.10 | 0.28 |

*Table S 7 Change in accuracy (*$\Delta_{\rho}$*) and response to selection in CB performance (*$\Delta_{R}$*) when the breed-origin of alleles (BOA) in crossbreds is considered, compared to when the BOA is ignored.*

| study | data | trait | line^1^ | model | r_pc_ | N_PB_ | N_CB_ | $\Delta_{\rho}$ | $\Delta_{R}$ |
| --- | --- | --- | --- | --- | --- | --- | --- | --- | --- |
| Ibanez-Escriche2009 | stoch | T1 | 3w dam | A-BOA | 1 |  | 4000 | 0.02 |  |
| Ibanez-Escriche2009 | stoch | T1 | 3w sire line | A-BOA | 1 |  | 4000 | *-0.03* |  |
| Ibanez-Escriche2009 | stoch | T1 | 4w PB | A-BOA | 1 |  | 4000 | 0.01 |  |
| Ibanez-Escriche2009 | stoch | T1 | 3w sire line | A-BOA | 1 |  | 4000 | *-0.06* |  |
| Kinghorn2010 | stoch | T1 |  | A-BOA |  |  | 400 |  | 0.03 |
| Zeng2013 | stoch | High-D |  | A-BOA |  |  | 1000 |  | 0.00 |
| Zeng2013 | stoch | High-D |  | A-BOA vs A-D |  |  | 1000 |  | *-0.02* |
| Zeng2013 | stoch | Real-D |  | A-BOA |  |  | 1000 |  | *-0.01* |
| Zeng2013 | stoch | Real-D |  | A-BOA vs A-D |  |  | 1000 |  | *-0.02* |
| Zeng2013 | stoch | No-D |  | A-BOA |  |  | 1000 |  | *-0.06* |
| Zeng2013 | stoch | No-D |  | A-BOA vs A-D |  |  | 1000 |  | *-0.05* |
| Esfandyari2015a^2^ | stoch | T1 | PB line | A-D-BOA | 0.78 |  | 2000 |  | 0.02 |
| Esfandyari2015a | stoch | T2 | PB line | A-D-BOA | 0.78 |  | 500 |  | *-0.01* |
| Esfandyari2015a | stoch | T3 | PB line | A-D-BOA | 0.78 |  | 8000 |  | 0.03 |
| Sevillano2017 | pig | ADG | PB LL | A-BOA | 0.3 | 1750 | 1400 | 0.06 |  |
| Sevillano2017 | pig | ADG | PB sires | A-BOA | 0.52 | 1900 | 1200 | 0.01 |  |
| Sevillano2017 | pig | BF | PB LL | A-BOA | 0.7 | 1750 | 1400 | 0.00 |  |
| Sevillano2017 | pig | BF | PB LW | A-BOA | 0.62 | 5000 | 1200 | *-0.02* |  |
| Sevillano2017 | pig | BF | PB sires | A-BOA | 0.69 | 1900 | 1200 | 0.00 |  |
| Sevillano2017 | pig | LDP | PB LL | A-BOA | 0.56 | 1750 | 1400 | *-0.01* |  |
| Sevillano2017 | pig | LDP | PB LW | A-BOA | 0.62 | 4000 | 1200 | *-0.02* |  |
| Sevillano2017 | pig | LDP | PB sires | A-BOA | 0.55 | 1900 | 1200 | *-0.01* |  |
| Sevillano2019 | pig | ADG | PB LL | A-BOA | 0.44 | 3228 | 2816 | 0.01 |  |
| Sevillano2019 | pig | ADG | PB sires | A-BOA | 0.66 | 7575 | 2816 | 0.00 |  |
| Sevillano2019 | pig | ADG | PB LW | A-BOA | 0.49 | 12794 | 2816 | 0.01 |  |
| Duenk2019 | brl | BW35 | PB sires | A-BOA | 0.96 |  | 4445 | *-0.04* |  |
| Duenk2019 | brl | BW7 | PB sires | A-BOA | 0.8 |  | 4655 | 0.04 |  |

*^1^UR = unrelated parental lines
^2^ The model that considers the BOA is written as a model that accounts for imprinting effects. But in these simulations, they are (probably) equivalent (there was no epistasis). However, the BOA model may suffer from reduced power and deviations in genotype probabilities from expectation, while the imprinting model may not.*

*Table S 8 Change in accuracy (*$\Delta_{\rho}$*) and response to selection in CB performance (*$\Delta_{R}$*) when the breed-origin of alleles (BOA) in crossbreds is considered, compared to when CB genotypes are not collected.*

| study | data | trait | line^1^ | r_pc_ | N_PB_ | N_CB_ | a_PB_ | a_CB_ | $\Delta_{\rho}$ | $\Delta_{R}$ |
| --- | --- | --- | --- | --- | --- | --- | --- | --- | --- | --- |
| Ibanez-Escriche2009 | stoch | T1 | 3w dam | 1 | 4000 | 4000 | 0 | 0 | *-0.07* |  |
| Ibanez-Escriche2009 | stoch | T1 | 3w sire | 1 | 4000 | 4000 | 0 | 0 | *-0.14* |  |
| Ibanez-Escriche2009 | stoch | T1 | 4w PB | 1 | 4000 | 4000 | 0 | 0 | *-0.08* |  |
| Ibanez-Escriche2009 | stoch | T1 | 4w PB UR^1^ | 1 | 4000 | 4000 | 0 | 0 | *-0.32* |  |
| Kinghorn2010 | stoch | T1 |  |  | 400 | 400 |  |  |  | 0.10 |
| Esfandyari2015a | stoch | T1 | PB line | 0.78 | 2000 | 2000 | 0-0.5 | 0-0.125 |  | 0.10 |
| Xiang2016b | pig | TNB | LL sires | 0.79 | 7800 | 5200 | 0-1 | 0.50 | 0.07 |  |
| Xiang2016b | pig | TNB | LL sires | 0.79 | 7800 | 5200 | 0-1 | 0.50 | 0.04 |  |
| Xiang2016b | pig | TNB | YY sires | 0.68 | 7800 | 5200 | 0-1 | 0.50 | 0.09 |  |
| Xiang2016b | pig | TNB | YY sires | 0.68 | 7800 | 5200 | 0-1 | 0.50 | 0.07 |  |
| Lopes2017 | pig | GLE-LL | CB | 0.9 | 832 | 832 | 0 | 0 | 0.23 |  |
| Lopes2017 | pig | GLE-LW | CB | 0.9 | 832 | 832 | 0 | 0 | 0.11 |  |
| Lopes2017 | pig | LS-LL | CB | 0.9 | 832 | 832 | 0 | 0 | 0.16 |  |
| Lopes2017 | pig | LS-LW | CB | 0.9 | 832 | 832 | 0 | 0 | 0.17 |  |
| Duenk2019 | broilers | BW35 | PB sire line | 0.96 | 4471 | 4445 | 0-0.125 | 0-0.125 | *-0.14* |  |
| Duenk2019 | broilers | BW7 | PB sire line | 0.8 | 4687 | 4655 | 0-0.125 | 0-0.125 | 0.04 |  |
| Wientjes2020 | pigs | T1 | 2CB | 0.75 | 2400 | 2400 | 0-0.5 | 0-0.0625 | 0.10 |  |
| Wientjes2020 | pigs | T1 | 2CB_1MP | 0.75 | 2400 | 2400 | 0-0.5 | 0-0.0156 | 0.08 |  |
| Wientjes2020 | pigs | T1 | 4CB | 0.75 | 2400 | 2400 | 0-0.5 | 0-0.0156 | *-0.01* |  |
| Wientjes2020 | pigs | T1 | 4CB_1MP | 0.75 | 2400 | 2400 | 0-0.5 | 0-0.004 | *-0.03* |  |
| Wientjes2020 | pigs | T1 | 2CB | 0.75 | 9600 | 9600 | 0-0.5 | 0-0.0625 | 0.16 |  |
| Wientjes2020 | pigs | T1 | 2CB_1MP | 0.75 | 9600 | 9600 | 0-0.5 | 0-0.0156 | 0.13 |  |
| Wientjes2020 | pigs | T1 | 4CB | 0.75 | 9600 | 9600 | 0-0.5 | 0-0.0156 | 0.01 |  |
| Wientjes2020 | pigs | T1 | 4CB_1MP | 0.75 | 9600 | 9600 | 0-0.5 | 0-0.004 | *-0.01* |  |
| Wientjes2020 | pigs | T2 | 2CB | 0.5 | 2400 | 2400 | 0-0.5 | 0-0.0625 | 0.28 |  |
| Wientjes2020 | pigs | T2 | 2CB_1MP | 0.5 | 2400 | 2400 | 0-0.5 | 0-0.0156 | 0.27 |  |
| Wientjes2020 | pigs | T2 | 4CB | 0.5 | 2400 | 2400 | 0-0.5 | 0-0.0156 | 0.16 |  |
| Wientjes2020 | pigs | T2 | 4CB_1MP | 0.5 | 2400 | 2400 | 0-0.5 | 0-0.004 | 0.16 |  |
| Wientjes2020 | pigs | T2 | 2CB | 0.5 | 9600 | 9600 | 0-0.5 | 0-0.0625 | 0.39 |  |
| Wientjes2020 | pigs | T2 | 2CB_1MP | 0.5 | 9600 | 9600 | 0-0.5 | 0-0.0156 | 0.36 |  |
| Wientjes2020 | pigs | T2 | 4CB | 0.5 | 9600 | 9600 | 0-0.5 | 0-0.0156 | 0.24 |  |
| Wientjes2020 | pigs | T2 | 4CB_1MP | 0.5 | 9600 | 9600 | 0-0.5 | 0-0.004 | 0.22 |  |

*^1^UR = unrelated parental lines*
